# Boosting mRNA cancer vaccine efficacy via targeting *Irg1* on macrophages in lymph nodes

**DOI:** 10.7150/thno.110305

**Published:** 2025-05-25

**Authors:** Wenwen Wei, Xiao Yang, Yeshan Chen, Mengjie Che, Ying Ye, Yue Deng, Mengyao Su, Yajie Sun, Jingshu Meng, Yan Hu, Jiacheng Wang, Yijun Wang, Zishan Feng, Zhiyuan Zhou, Yan Li, Qian Li, Zhanjie Zhang, Bian Wu, Haibo Zhang, You Qin, Lu Wen, Chao Wan, Kunyu Yang

**Affiliations:** 1 Cancer Center, Union Hospital, Tongji Medical College, Huazhong University of Science and Technology, Wuhan 430022, China; 2 Institute of Radiation Oncology, Union Hospital, Tongji Medical College, Huazhong University of Science and Technology, Wuhan 430022, China; 3 Hubei Key Laboratory of Precision Radiation Oncology, Wuhan 430022, China; 4 Cancer Center, Department of Radiation Oncology, Zhejiang Provincial People's Hospital (Affiliated People's Hospital), Hangzhou Medical College, Hangzhou, Zhejiang 310000, China

**Keywords:** taconate, mRNA cancer vaccines, macrophages, DCs, anti-PD-1 antibody

## Abstract

**Rationale:** mRNA cancer vaccines show great promise for tumor therapy, but the therapeutic efficacy is limited. Metabolites play critical roles in immunomodulation. However, their role in mRNA cancer vaccines remains poorly understood.

**Methods:** Metabolome analysis and single-cell RNA sequence were performed to explore the most important metabolite and its source cell. B16-F10-OVA-bearing wide-type and *Irg1*-depleted C57BL/6 mice were treated with OVA-LNP, OVA&si-Irg1-LNP, or anti-PD-1 antibody to evaluate therapeutic efficacy. Flow cytometry analysis was used to examine the immune cells within the lymph nodes, spleens, and the tumor immune environment.

**Results:** We found that macrophage-derived itaconate was increased markedly in activated ipsilateral lymph nodes after ovalbumin-encoding mRNA-lipid nanoparticle (OVA-LNP) injection, compared to homeostatic contralateral lymph nodes. Depleting the immune-responsive gene 1(*Irg1*), which encodes the itaconate-production enzyme aconitate decarboxylase (ACOD1), in macrophages improved dendritic cell antigen presentation and enhances T cell function. Combining *Irg1* knockdown via small interfering RNA (siRNA) with OVA mRNA in LNPs augmented the therapeutic efficacy of mRNA cancer vaccines, both as monotherapy and in combination with an anti-programmed cell death-1 antibody.

**Conclusions:** Our findings reveal a link between itaconate and mRNA cancer vaccines, suggesting that targeting *Irg1 via* siRNA-LNP could be a promising strategy to improve the therapeutic efficacy of mRNA cancer vaccines.

## Introduction

The first mRNA cancer vaccine, encoding carcinoembryonic antigen (CEA), was shown to induce an immune response to CEA-expressing tumor cells in mice in 1995^1^. Since then, various *in vitro* preclinical studies of mRNAs have been conducted, revealing the therapeutic potential of mRNA-based approaches. Compared to previous DNA-based therapeutics, mRNA-based therapies have the advantage of enabling efficient expression while reducing the risk of insertional mutagenesis and stable integration of DNA into the host genome 2. However, challenges such as the inherent instability and high immunogenicity of naked mRNA, lack of suitable manufacturing methods, and inefficient delivery methods have hindered their advancement for a long time. The success of mRNA vaccines against COVID-19 has advanced mRNA technology and generated interest in mRNA-based tumor therapeutics 3, 4. Chemical modification, product purification, and sequence optimization have been attempted to enhance mRNA stability, prolong translation, and reduce immunogenicity 5-7. In addition, mRNA-packaging nanotechnologies, such as encapsulating mRNA into nanoparticles, can protect mRNA from RNase-mediated degradation in cellular fluids and facilitate efficient delivery into cells and organs for subsequent translation. For instance, the current research focus on lipid nanoparticles (LNPs) has demonstrated effective mRNA delivery against COVID-19 in the clinic 3. Thus, several preclinical and clinical studies have been conducted to explore the effectiveness and safety of mRNA cancer vaccines, either alone or in combination with chemotherapy or immunotherapy 8. In some clinical studies, mRNA-LNPs have demonstrated considerable potential for cancer therapy 9, 10. However, some studies on mRNA cancer vaccines have either not shown any benefits or suggested harmful effects 11, 12. Thus, further research is required to understand the interaction between mRNA-LNPs and immune cells, and to optimize their efficacy and safety.

Accumulating evidence indicates that metabolic reprogramming hinders the ability of immune cells to initiate an effective anti-tumor immune response 13. Many metabolites serve dual roles in metabolism and signaling, exerting direct or indirect immunomodulatory effects. For instance, lactate, which accumulates in large quantities within solid tumors, inhibits the proliferation and activation of cytotoxic T lymphocytes (CTLs) and natural killer (NK) cells 14, 15. It also supports the immunosuppressive and tumor-promoting functions of CD4^+^ CD25^+^ FOXP3^+^ regulatory T (Treg) cells 16, 17, and promotes the polarization of tumor-associated macrophages (TAMs) towards an immunosuppressive M2-like phenotype, which is associated with poor disease outcomes 18, 19. Moreover, immune cells- including T cells 20, 21, dendritic cells (DCs) 22, and Treg cells 23 take up fatty acids in a CD36-dependent manner. This process directly suppresses the function of CTLs and DCs, while promoting the expansion or activity of Treg cells. In summary, most metabolites, which are produced during the immune response, suppress immune cells such as T cells, DCs, and TAMs or activate immunosuppressive cells to influence the function of immune cells.

The mRNA encodes one or more proteins that are taken up by antigen-presenting cells (APCs), such as DCs and macrophages in lymph nodes, and presented on the surface by major histocompatibility complexes (MHCs) to induce anti-tumor immunity. Based on the direct or indirect impact of metabolites on the function or quantity of immune cells, we hypothesized that mRNA cancer vaccines, by inducing certain metabolites, would affect the function of immune cells. Thus, we analyzed the metabolome in the contralateral (non-draining; cLN) and ipsilateral (draining; iLN) lymph nodes (LNs) after subcutaneous injection of ovalbumin (OVA)-encoding mRNA-LNP (OVA-LNP). These findings indicated that the metabolite profile was different between iLNs and cLNs, and itaconate was the most highly upregulated metabolite in iLNs compared to cLNs. Itaconate is produced *via* the decarboxylation of the tricarboxylic acid (TCA)-derived cis-aconitate by aconitate decarboxylase (ACOD1), encoded by immune-response gene1 (*Irg1*, also known as *Acod1*) 24. Previous studies have demonstrated that itaconate rapidly accumulates to high levels in myeloid cells during both infectious and sterile inflammatory conditions, where it plays an immunomodulatory role in host innate immunity against infectious pathogens and non-infectious inflammatory processes 25. However, whether itaconate affects mRNA vaccine efficacy remains unclear. In this study, we demonstrated that macrophage-derived itaconate in LNs diminishes antigen presentation of DCs, and T cell functions, which decreases the efficacy of mRNA cancer vaccines. Moreover, we designed the OVA&si-*Irg1*-LNP, which contained OVA mRNA and *Irg1* small interfering (si)RNA (si-*Irg1*), suppressed* Irg1* expression, and itaconate secretion *in vitro* and *in vivo.* More importantly, OVA&si-*Irg1-*LNP enhanced anti-tumor efficacy alone or combined with anti-programmed cell death-1 (anti-PD-1) antibody and showed good safety.

## Materials and Methods

### Mice

All animal experiments followed the guidelines of the Hubei Provincial Animal Care and Use Committee and the Animal Experimentation Ethics Committee of Huazhong University of Science and Technology. Female C57BL/6 mice aged 6-8 weeks were purchased from Hunan Slyke Jingda Laboratory Animal Co. LTD. The mice, including C57BL/6NJ-* Irg1*^em1(IMPC)^J/J mice (also known as *Irg1*^-/-^), Myeloid cell-specific *Irg1*-deficient (*Irg1*^f/f^Lyz2^Cre^ mice), were bred and kept in specific-pathogen-free conditions at the Animal Center of Huazhong University of Science and Technology (HUST) in Wuhan, China. They were housed in a 12/12 h dark/light cycle environment with appropriate temperature, humidity, and adequate food and water.

### Cell lines and cell culture

Mouse B16-F10 cells were acquired from the China Center for Type Culture Collection (Wuhan, China). The B16-F10-OVA cell line, which is stably transfected with ovalbumin, was kindly provided by prof. Bo Huang at the Chinese Academy of Medical Sciences and Peking Union Medical College, Beijing. The cells were grown in RPMI 1640 medium (11875093, Gibco) with 10% fetal bovine serum (FBS, 164210-50, Procell) and 1% penicillin/streptomycin (5140122, Gibco).

### Metabolome analysis

Metabolome analysis was performed as described previously 26. In brief, fresh iLNs and cLNs were collected from C57BL/6 mice at 24 h post-subcutaneous injection of OVA-LNP into the right flank. The LNs were homogenized in 70% methanol and centrifuged to obtain the supernatant. Next, acetonitrile (with 0.1% formic acid) and ultrapure water (with 0.1% formic acid) were added to the supernatant. After another round of centrifugation and filtration using a Waters ACQUITY UPLC HSS T3 Column, the concentrated filtrate was used for metabolite analysis.

### Single-cell RNA sequencing

Three C57BL/6 mice received a subcutaneous injection of 5 μg of OVA-LNP. After 24 h, total iLNs and cLNs were collected and preserved in MACS Tissue Storage Solution (130-100-008, Miltenyi Biotec). The samples were then centrifuged at 50 g for 1 min at 4 °C and digested using a digestion solution (130-095-929, Miltenyi Biotec) in a 37 °C water bath for 45 min. After the incubation, the solution was filtered using a 40-μm cell strainer (CLS431750, Corning), and the red blood cells were removed with red blood cell lysis buffer (11814389001, Roche). Finally, the single cells were resuspended in PBS with 0.01% Bovine serum albumin (A1933, Sigma) and subjected to single-cell RNA sequencing (scRNA-seq) using MobiDrop (Zhejiang) Co., Ltd., China.

### *In vitro* stimulation of bone marrow-derived macrophages (BMDMs)

The bone marrow-derived cells from female C57BL/6 or *Irg1*^-/-^ mice aged 6-10 weeks were collected, centrifuged for 5min, and depleted of red blood cells using a lysis buffer. Then, the femoral and tibial specimens were cultured in RPMI 1640 supplemented with 10% FBS and 20 ng/mL macrophage colony-stimulating factor (M-CSF, 576406, Biolegend). On day 6, the BMDMs were stimulated by 0.3 μg/mL OVA-LNP, and samples were collected at 0, 2, 4, 8, 12, and 24 h for quantitative reverse transcription-polymerase chain reaction (qRT-PCR) and flow cytometry. The supernatant at 24 h was collected as a conditioned medium.

### Flow cytometry

*In vitro*, BMDMs and bone marrow-derived DCs (BMDCs) were collected and stained with antibodies at 4 ℃ for 30 min. And stained samples were acquired on the BD FACSymphony flow cytometer (BD Biosciences). Antibodies used in this study were listed in Supplementary Table 1.

*In vivo*, lymph nodes and tumors were harvested at the indicated time and then digested by 0.5 mg/mL hyaluronidase (HY-E70182, MedChemExpress) and 0.32 mg/mL collagenase V (HY-E70005E, MedChemExpress) for 45 min at 37 ℃ to make single-cell suspensions. Spleens and blood were depleted red blood cells with lysis buffer to obtain the single cells. The single-cell suspension was blocked with anti-mouse CD16/32 (101320, Biolegend), and dead cells were stained with zombie NIR^TM^ (423106, Biolegend) or Violet^TM^ (423114, Biolegend) dye for 30 min. For cell membrane protein staining, cells were stained at 4 ℃ for 30 min. For intracellular cytokine staining, cells were incubated with a stimulation cocktail (1ug/mL Ionomycin (HY-13434, MedChemExpress), 1.5 μg/mL Monensin (HY-N4302, MedChemExpress), and 100 ng/mL Phorbol 12-myristate 13-acetate (HY-18739, MedChemExpress)) for 4 h or 2 μM OVA peptide (HY-P3715, MedChemExpress) for 16 h before cell surface and cytokine staining. Then, the cells were fixed, permeabilized, and stained with antibodies. All samples were analyzed using the BD FACSymphony flow cytometer from BD Biosciences. Antibodies used in this study were listed in Supplementary Table 1.

### *In vivo* anti-tumor assay

For *in vivo* experiments, 6-8 weeks WT and *Irg1*^-/-^ mice were used, and 3×10^5^ B16-F10-OVA cells were implanted subcutaneously at the right flank on day 0. When the tumor volume reached 50mm^3^, mice were randomly assigned to different groups and were administered the first dose of vaccination (5 μg OVA-LNP, si-*Irg1*-LNP, or OVA&si-*Irg1*-LNP) subcutaneously on day 7 and the booster dose on day 12. Mice with LNP without any mRNA are used as the control group. The mice were also administered InVivoMAb anti-mouse PD-1 (CD279, Clone: RMP1-14) (10 mg/kg) (BE0146, Bioxcell) intraperitoneally for the immune checkpoint blockade combined therapy on days 9, 14, and 17. To evaluate the effect of itaconate on tumors, 4-OI (50 mg/kg) was administered intraperitoneally daily from day 8 to day 14. The tumor volume (Vs) was monitored by a Vernier caliper and calculated as V=L (length) x W (Width)^2^/2. Mice were sacrificed if the average diameter exceeded 15 mm or the tumor volumes exceeded 2000 mm^3^.

### qRT-PCR

Total RNA was extracted from cells or Tissues using RNAiso Plus Reagent (9109, Takara). After measurement for concentration and purity, RNA was reverse-transcribed into cDNA with HiScript III 1st Strand cDNA Synthesis Kit (+gDNA wiper) (R312-01, Vazyme). qRT-PCR was performed using ChamQ SYBR qPCR Master Mix (Q311-02, Vazyme). The primers used in the study are listed in Supplementary Table 2.

### LC/MS analysis

The supernatant from BMDM or BMDC after stimulation with LNPs for 24 h, and the LNs 24 h after a subcutaneous injection of 5 μg LNPs was collected for LC/MS analysis.

### Synthesis and formulation of mRNA-encapsulating LNPs

The LNPs were commercially synthesized by Rhegen Bio. Inc. First, OVA mRNA, si-Ctrl (Sequence-F: UUCUCCGAACGUGUCACGUTT; Sequence-R: ACGUGACACGUUCGGAGAATT), and si-*Irg1* (F: CAGGUUUACCAAUAUCUAAUU; R: UUAGAUAUUGGUAAACCUGGG) siRNA were diluted in 100 mM citrate buffer (pH 4.00) as the aqueous phase. At the same time, PEG-lipid (ALC-0519, 1.6%), cationic lipid (DSPC, 9%), cholesterol (42.7%), and phospholipid (ALC-0315, 46.3%) were dissolved in ethanol as the lipid phase. The aqueous and lipid phases were mixed at a flow rate of 3: 1 using a Liposyn X Intelligent Lipid Nanoparticle Synthesis System (ED1001, Enoch (Shenzhen) Biotechnology Co). Next, the samples were transferred to a 100 kDa dialysis bag for overnight dialysis and subsequently concentrated using an ultrafiltration centrifuge tube. Finally, the LNPs underwent quality assurance testing for particle size, potential, and encapsulation rate.

### *In vivo* safety evaluation

To evaluate the safety of si-*Irg1*-LNP, OVA-LNP, and OVA&si-*Irg1*-LNP *in vivo*, the body weight was monitored every 3 days for 21 days after subcutaneous injection of 5 μg LNPs. Blood serum aspartate transaminase (AST), alanine aminotransferase (ALT), blood urea nitrogen (BUN), and creatinine (CR) levels were measured on days 7, 14, and 21. Additionally, heart, liver, spleen, lung, and kidney tissue samples were collected at the end of the experiment for histological and aminotransaminase analysis.

### Statistical analysis

We used unpaired two-tailed *Student's* t-test and one-way ANOVA to compare two or more groups. Survival analysis was conducted with the log-rank test. All the results were analyzed using GraphPad Prism software and are presented as means ± SD or means ± SEM. The flow cytometry data was analyzed using FlowJo. Significant differences were indicated: ns = no significance, * *p* < 0.05, ** *p* < 0.01, *** *p* < 0.001.

## Results

### Metabolome analysis identified that itaconate suppresses T cell response to mRNA cancer vaccine

**Figure 1:**
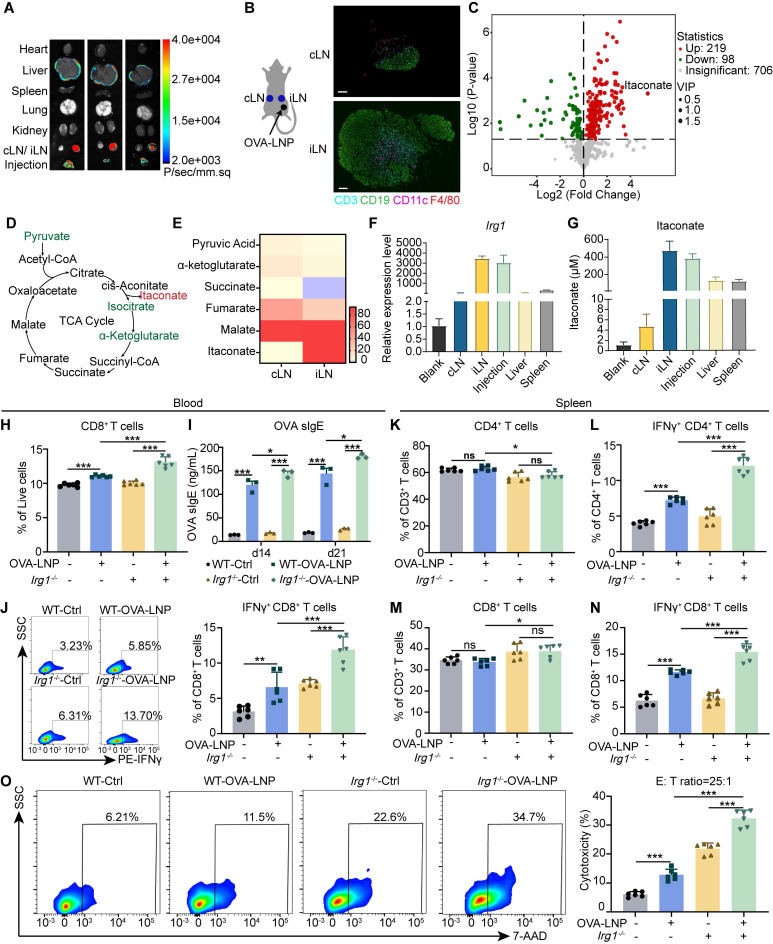
OVA-LNP-induced itaconate suppressed T cell response. (A) The luminescence image of organs in mice after Luciferase-LNP injection for 24h. (B-E) Mice were injected with 5 μg OVA-LNP subcutaneously, and iLNs and cLNs were obtained for metabolome analysis and immunofluorescence at 24 h, n = 5. (B) Immunofluorescence of T cells (CD3), B cells (CD19), DCs (CD11c), and Macrophages (F4/80). scale bars, 100 μm. (C) Volcano plot of different metabolites between iLNs and cLNs. (D) The schematic of the TCA cycle. (E) The heatmap of metabolites of the TCA cycle in iLNs compared to cLNs. (F) *Irg1* mRNA expression of organs in mice after OVA-LNP injection for 24h, n = 3. (G) Itaconate concentration of organs in mice after OVA-LNP injection for 24h, n = 3. (H-O) T-cell and B-cell responses to OVA-LNP in WT and *Irg1*^-/-^ mice at days 14 and 21. (H) The proportion of CD8^+^ T cells in blood, n = 6. (I) The concentration of OVA sIgE in blood serum was detected by ELISA, n = 3. (J) The percentage of IFNγ^+^ CD8^+^ T cells in blood was detected by flow cytometry, n = 6. (K-N) The proportion of CD4^+^(K), IFNγ^+^ CD4^+^(L), CD8^+^(M), IFNγ^+^ CD8^+^ (N)T cells in CD3^+^ T cells isolated from spleens h, n = 6. (O) Cytotoxicity of CD3^+^ T cells isolated from spleens after being incubated with B16-F10-OVA tumor cells, n = 6. ns = no significance, * p < 0.05, ** p < 0.01, *** p < 0.001.

Luciferase-LNPs were injected subcutaneously into mice, and organs were harvested for bioluminescence imaging after 24 h 27-29. The iLN exhibited the highest bioluminescence signal (**Figure 1A**). Then, cLNs and iLNs were collected from mice that received a subcutaneous injection of 5 μg OVA-LNP, and the iLNs exhibited an activated status compared to the homeostatic cLNs (**Figure 1B**). Water-soluble metabolites analysis indicated that OVA-LNP strongly influenced the immunized LN metabolic landscape, with iLNs being distinct from cLNs (**Figure S1A**). In total, 219 metabolites were upregulated and 98 metabolites were downregulated after LNP injection, with itaconate being the most upregulated (**Figure 1C**). Pathway analysis of significantly different metabolites between iLNs and cLNs showed that pyrimidine, galactose, glycerophospholipid, and purine metabolism, and the TCA cycle were strongly associated with immune activation (**Figure S1B**). We analyzed the metabolites involved in the TCA cycle and observed that itaconate was upregulated by 44.6-fold. Additionally, its upstream and downstream metabolites, such as pyruvate, isocitrate, and α-ketoglutarate, were downregulated as expected (**Figure 1D-E**, **S1C-D**). Furthermore, we observed the downregulation of serine and glycine in iLNs compared to that in cLNs (**Figure S1E**). A previous study indicated that itaconate suppresses CD8^+^ T cell proliferation and activation by hindering aspartate, serine, and glycine biosynthesis 30. Thus, we speculated that itaconate induced by OVA-LNP may affect T cell function. Furthermore, *Irg1* expression and itaconate release were measured in various organs after OVA-LNP injection. The results demonstrateded that *Irg1* and itaconate were predominantly upregulated mainly in iLNs (**Figure 1F-G**).

Because ACOD1 is a critical enzyme for itaconate production, we used *Irg1*-deficient mice (**Figure S1F**) to assess the effect of itaconate on tumors. To assess the impact of itaconate on the anti-tumor efficacy of OVA-LNP, we administered 4-octyl itaconate (4-OI), a cell-permeable derivative of itaconate, to B16-F10 tumor-bearing *Irg1*^-/-^ mice. The results showed that 4-OI significantly increase tumor volume and weight after OVA-LNP treatment (**Figure S1F-H**). Meanwhile, 4-OI inhibited the expression of OVA and MHC I on DCs in iLNs (**Figure S1I-J**). Within the tumor microenvironment (TME), 4-OI reduced the frequencies of CD8^+^ and IFNγ^+^ CD8^+^ T cells (**Figure S1K-L**), but had no significant impact on CD4^+^, Foxp3^+^ CD4^+^ T cells, or myeloid-derived suppressor cells (MDSCs), including monocytic (M-MDSC) and granulocytic (M-MDSC) subsets (**Figure S1M-P**).

We next evaluate the T cell response to OVA-LNP in the *Irg1*-deficient mice (*Irg1*^-/-^) (**Figure S2A**). First, *Irg1* and itaconate levels were measured in both WT and *Irg1*^-/-^ mice. While *Irg1* deficiency did not affect the bioluminescence distribution (**Figure S2B**), *Irg1* expression and itaconate levels were significantly reduced in the peripheral blood and organs of *Irg1*^-/-^ mice (**Figure S2C-E**). Subsequently, T cell function was assessed in the peripheral blood and spleen following the injection of 5 μg OVA-LNP (**Figure S2F**). The results indicated an increase in CD8^+^ T cells in the blood after restimulation with 2 μM OVA peptide in the OVA-LNP immunized group (**Figure 1H**). Additionally, there was higher secretion of OVA sIgE and sIgG at days 14 and 21 (**Figure 1I, S2G**), and an elevated presence of interferon-γ (IFNγ^+^) CD8^+^ T cells in the blood at day 21 (**Figure 1J**) compared to the control group. Notably, *Irg1* deficiency further enhanced these effects (**Figure 1H-J**). We also examined CD4^+^ and CD8^+^ T cells in the spleens of the control and OVA-LNP immunized mice. The percentages of CD4^+^ and CD8^+^ T cells among CD3^+^ T cells were unaffected by OVA peptide stimulation (**Figure 1K, M**). However, there was a significant increase in IFNγ^+^ CD4^+^ and IFNγ^+^ CD8^+^ T cells in the OVA-LNP immunized group, and this increase was even more pronounced in mice with *Irg1* deficiency (**Figure 1L, N**, **S2H**). Furthermore, when spleen cells were co-cultured with B16-F10-OVA cells at an effector: target (E: T) ratio of 25: 1, the most robust cytotoxicity was observed in the OVA-LNP immunized *Irg1*-deficient group (**Figure 1O**). These data indicate that *Irg1* affects T cells following OVA-LNP immunization.

### OVA-LNP-induced itaconate derives from macrophages in iLNs

**Figure 2:**
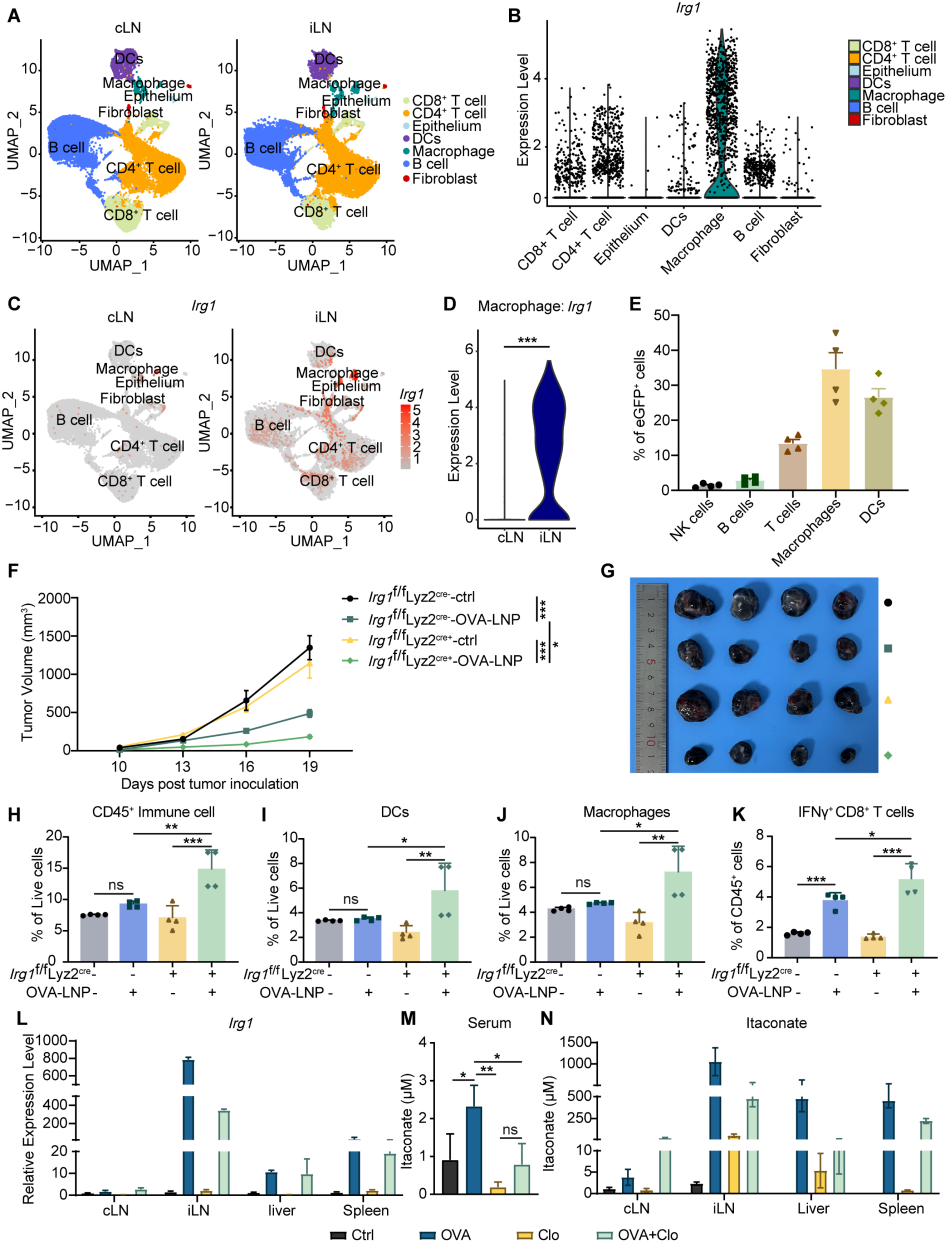
OVA-LNP-induced itaconate derived from macrophages in LNs. (A-D) The single-cell sequence of cLNs and iLNs was collected at 24 h after the OVA-LNP injection, n = 5. (A) The UMAP visually represented different immune cell types in cLN and iLN. (B) *Irg1* expression levels of different immune cells in LNs. (C) The UMAP plot depicted the expression levels of *Irg1* in cLNs and iLNs. (D) Comparing the *Irg1* expression levels of macrophages in cLNs and iLNs. (E) The different types of immune cells in iLNs, such as NK cells (NK1.1), B cells (CD19), T cells (CD3), Macrophages (F4/80), and DCs (CD11c), were detected using flow cytometry at 24 h after subcutaneous injection of eGFP-LNP. (F-K) The therapeutic effect and the TME remodeling of OVA-LNP in *Irg1*^f/f^ Lyz2 ^cre-^ (n = 4) and *Irg1*^f/f^ Lyz2 ^cre+^ (n = 4) mice. (F) Tumor growth curve of ctrl and two-dose 5 μg OVA-LNP treatment on days 7 and 12 in *Irg1*^f/f^ Lyz2 ^cre-^ and *Irg1*^f/f^ Lyz2 ^cre+^ mice. (G) Tumor image on day 21. (H to K) The percentage of CD45^+^ immune cells (H), DCs (I), macrophages (J), and IFNγ^+^ CD8^+^ T cells (K) within the TME between four groups. (L) *Irg1* mRNA expression of organs in mice with Clo at day 1 and OVA-LNP at day 2 for 24h, n = 3. (M-N) The concentration of itaconate in the blood (M) and organs (N) in mice with Clo at day 1 and OVA-LNP at day 2 for 24h, n = 3. ns = no significance, * p < 0.05, ** p < 0.01, *** p < 0.001.

Itaconate is produced by myeloid cells, significantly activated macrophages, in response to inflammatory stimuli and cellular stresses. Recent studies have shown that *Irg1* is highly expressed in tumor-infiltration neutrophils (TINs), which constrains breast cancer metastasis 31. Thus, we investigated which cell type upregulated *Irg1* expression induced by OVA-LNP in iLNs. We dissociated iLNs and cLNs according to the procedure shown in Figure 1A and subjected them to scRNA-seq. The seven identified clusters were visualized using uniform manifold approximation and projection for reduction (UMAP) algorithm based on gene expression, including CD8^+^ T cell, CD4^+^ T cell, epithelium, DC, macrophage, B cell, and fibroblast (**Figure 2A**). The data revealed that *Irg1* was expressed in seven clusters and was highly expressed in macrophages (**Figure 2B**). Compared with cLNs, *Irg1* was significantly upregulated in iLNs, particularly in macrophages (**Figure 2C-D**). Additionally, we subcutaneously injected enhanced green fluorescent protein (eGFP)-LNP into C57BL/6 mice and obtained iLNs after 24 h. The percentage of eGFP^+^ immune cells was detected using flow cytometry, and the data indicated that macrophages were the primary eGFP^+^ cells among T cells, B cells, NK cells, and DCs (**Figure 2E**), which is consistent with the scRNA-seq results.

To confirm the *Irg1*-derived cell type, we inoculated 3×10^5^ B16-F10-OVA tumor cells subcutaneously into *Irg1*^f/f^ Lyz2^ cre+^ and *Irg1*^f/f^ Lyz2^ cre-^ mice, which *Irg1* deficiency specifically in macrophages (**Figure S3A**), and treated them with OVA-LNP on day 7 when tumor volume reached to 50mm^3^, followed by a boost dose on day 12. The tumor volume was monitored every 3 days after treatment. The results indicated that a deficiency in *Irg1*, specifically in macrophages, enhanced the anti-tumor effectiveness of the mRNA cancer vaccines (**Figure 2F-G**). Flow cytometry results suggested that a deficiency of *Irg1* in macrophages elevated the number of CD45^+^ immune cells, macrophages, and DCs within the TME (**Figure 2H-J**). In addition, T cell function was significantly increased after OVA-LNP treatment in* the Irg1*^f/f^ Lyz2^ cre+^ group, presented as upregulation of IFNγ^+^ CD8^+^ T cells (**Figure 2K**). Furthermore, clodronate liposomes (Clo) were administered to deplete macrophages prior to OVA-LNP injection. Flow cytometry analysis confirmed successful macrophage depletion in the peripheral blood (**Figure S3B**). Clo treatment reduced *Irg1* expression and itaconate levels in the peripheral blood and organs, which were otherwise elevated by OVA-LNP stimulation. (**Figure 2L-N**). These results confirmed that itaconate induced by OVA-LNP was derived from macrophages and affected anti-tumor efficacy *via* immune cells.

### *Irg1* deletion promotes pro-inflammatory activation of macrophages

**Figure 3:**
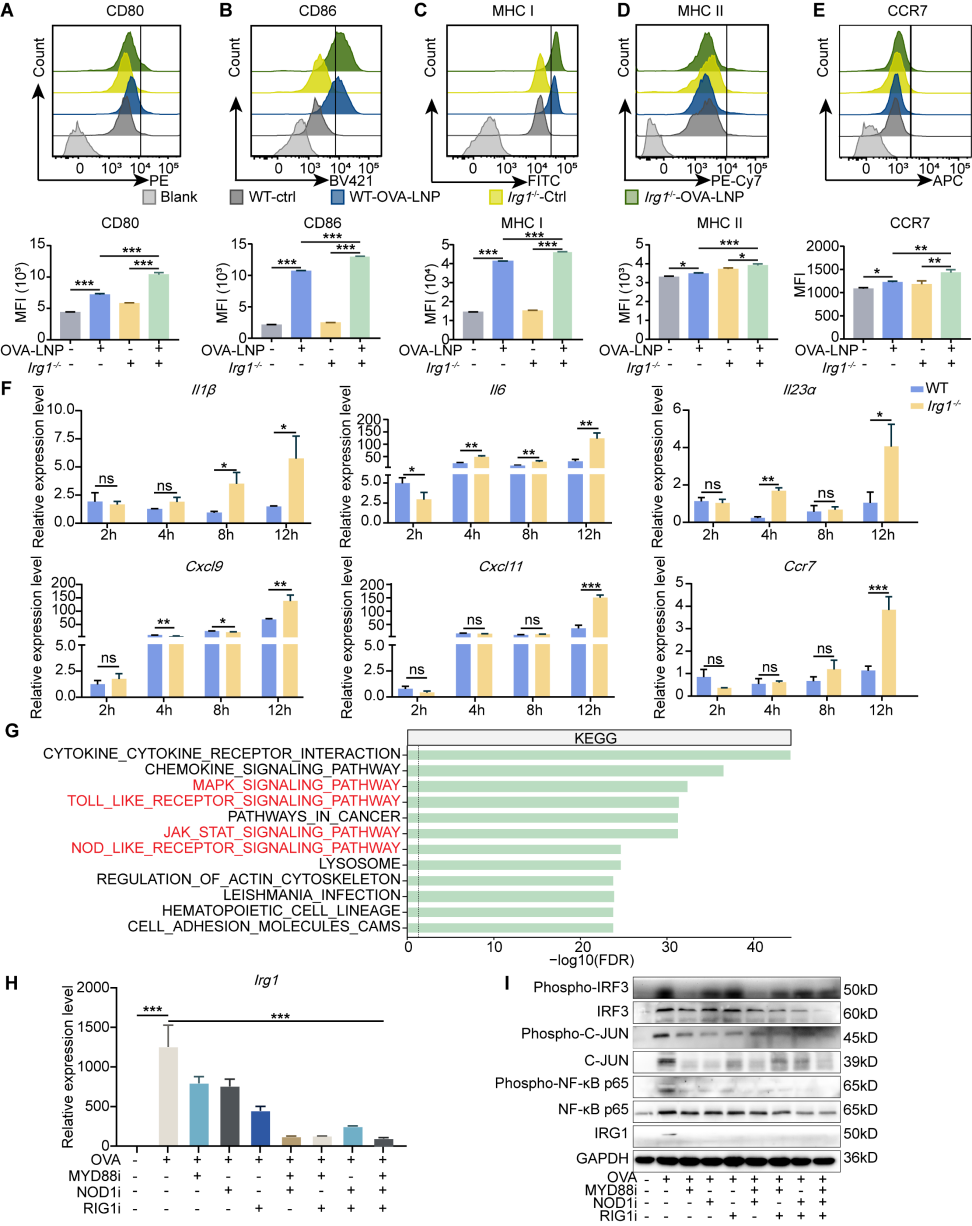
*Irg1*-induced by OVA-LNP suppressed the pro-inflammatory of macrophages. (A-E) The activation makers of WT and *Irg1^-/-^* macrophages were detected by flow cytometry at 24 h after being stimulated by 0.3 μg/mL ctrl-LNP or OVA-LNP. CD80 (A), CD86 (B), MHC I (C), MHC II (D), and CCR7 (E) levels of macrophages, n = 3. (F) The *Il1β, Il6, Il23α, Cxcl9, Cxcl10*, and *Ccr7* expression levels of WT and *Irg1*^-/-^ macrophages, which were stimulated by 0.3 μg/mL ctrl-LNP or OVA-LNP at 2, 4, 8, 12 h, were measured by qRT-PCR, n = 3. (G) Kyoto Encyclopedia of Genes and Genomes (KEGG) enrichment of macrophages in iLNs compared to cLNs. (H) qRT-PCR measured expression levels of *Irg1* in BMDMs after treatment with 0.3 μg/mL OVA-LNP and 10 μM MYD88 inhibitor (MYD88i), 10 μM NOD1 inhibitor (NOD1i), and 500 nM RIG1 inhibitor (RIG1i) for 12 h, n = 3. (I) BMDMs were treated with 0.3 μg/mL OVA-LNP and 10 μM MYD88 inhibitor (MYD88i), 10 μM NOD1 inhibitor (NOD1i), and 500 nM RIG1 inhibitor (RIG1i) for 24 h, and cells were collected for western blot analysis. ns = no significance, * p < 0.05, ** p < 0.01, *** p < 0.001.

Macrophages that deplete *Irg1* are polarized towards a pro-inflammatory state after lipopolysaccharide (LPS) and IFNγ stimulation, leading to enhanced tumor suppression and increased survival 32. First, we analyzed scRNA-seq and found that OVA-LNP induces pro-inflammatory and antigen-presentation pathway enrichment of macrophages in iLNs compared to that in cLNs (**Figure S4A**). To confirm the role of *Irg1* in macrophage activation, we isolated BMDMs from C57BL/6 (WT) and *Irg1*^-/-^ mice. After stimulation with OVA-LNP for 24 h, pro-inflammatory activation of BMDMs was detected using flow cytometry. The results indicated a higher upregulation of CD80, CD86, MHC I, MHC II, and CCR7 expression in *Irg1*-deleted BMDMs than WT BMDMs following treatment with OVA-LNP (**Figure 3A-E**). Pro-inflammatory genes such as *Il1β, Il6, Il23α, Cxcl9, Cxcl11*, and *Ccr7* were detected at 2, 4, 8, and 12 h post-OVA-LNP stimulation using qRT-PCR. We observed an increase in the expression of these genes in *Irg1*-deleted BMDMs, especially after 12 h of stimulation (**Figure 3F**). OVA mRNA expression increased significantly at 2 h and remained elevated up to 12 h, and there was no significant difference between WT and *Irg1*^-/-^ BMDMs (**Figure S4B**). Additionally, *Irg1* was upregulated at 2 h and notably increased at 12 h in WT BMDMs, whereas no expression was observed in *Irg1*^-/-^ BMDMs (**Figure S4C**). Additionally, we encapsulated LNPs with another mRNA, eGFP, and found that eGFP-LNP treatment similarly induced *Irg1* upregulation and itaconate release without affecting the viability or proliferation of BMDMs (**Figure S4D-G**). These data demonstrated that *Irg1* plays a critical role in regulating the pro-inflammatory activation of macrophages without affecting OVA expression following OVA-LNP stimulation *in vitro*.

To investigate the potential mechanisms of *Irg1* upregulation in macrophages, we analyzed the Kyoto Encyclopedia of Genes and Genomes (KEGG) enrichment pathways of macrophages in iLNs compared to those in cLNs. The data showed that except activation pathways, such as the MAPK, JAK-STAT signaling pathways, and cytokine-cytokine receptor interaction, pattern recognition receptors (PRRs) that participate in vaccine adjuvant responses, such as TOLL-like receptors (TLR) and NOD-like receptors (NLR) 33, were more enriched in iLNs than in cLNs (**Figure 3G**). Studies have reported that mRNA and certain cationic lipids possess intrinsic adjuvant activity and potent immunostimulatory properties, enabling them to be recognized by PRRs of the innate immune system 8, 34, 35. Therefore, we incubated OVA-LNP-treated macrophages with the TLR inhibitor (MYD88i), NOD1 inhibitor (NOD1i), and another essential PRR-RIG-1-like receptor (RLR) inhibitor (RIG1i), although did not enrich in the KEGG enrichment pathway. We found that all inhibitors suppress OVA-LNP-induced *Irg1* expression, and combining two inhibitors enhanced this effect, with three inhibitors showing the most potent inhibition (**Figure 3H**). PRRs induce gene expression via phosphorylated activation of interferon regulatory Factor 3 (IRF3), nuclear factor kappa-B (NF-κB), and c-JUN. Thus, we detected IRG1, IRF3, NF-κB p65 and JUN, and their phosphorylation level by western blotting analysis. The results showed that OVA-LNP induced increased IRG1 expression and IRF3, NF-κB p65 and JUN activation, and MYD88i, NOD1i, and RIG1i inhibited this effect (**Figure 3I**). These data indicated that OVA-LNP led to an increase in *Irg1* expression in macrophages *via* multiple PRR pathways.

### Macrophage-derived itaconate suppresses antigen presentation of DC in LNs

**Figure 4:**
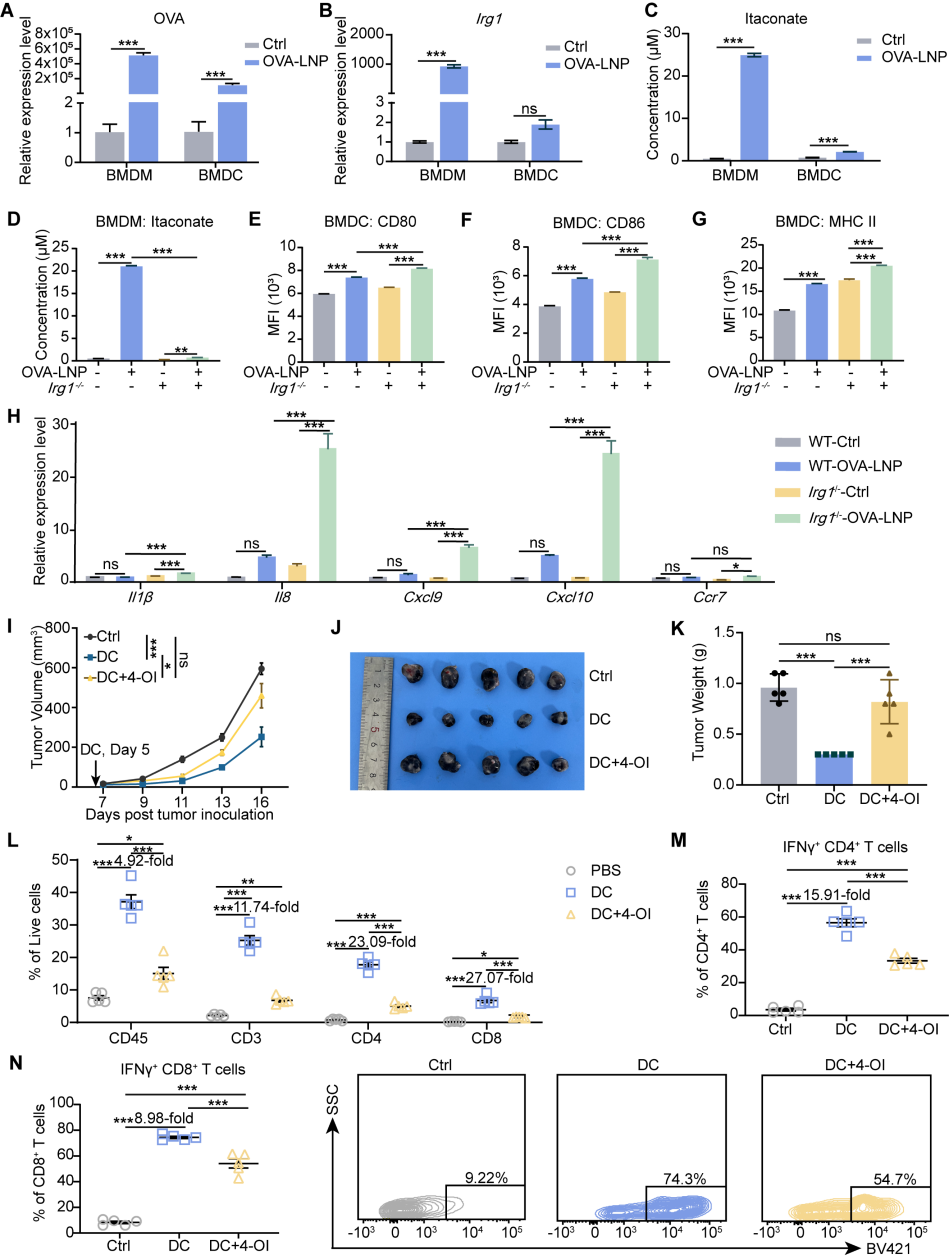
OVA-LNP-induced itaconate suppressed the function of DCs. (A-B) OVA (A) and *Irg1* (B) expression of BMDMs and BMDCs with 0.3 μg/mL OVA-LNP stimulation for 12 h, n = 3. (C) LC/MS measured the concentration of itaconate in the supernatant of BMDM and BMDC after 24 h of OVA-LNP stimulation, n = 3. (D) The concentration of itaconate in the WT and *Irg1*^-/-^ BMDM supernatant. (E-G) CD80 (E), CD86 (F), and MHC II (G) of BMDCs cultured with BMDMs CM for 24 h were measured by flow cytometry, n = 3. (H) The *Il1β, Il8, Cxcl9, Cxcl10*, and *Ccr7* expression levels of BMDC cultured with BMDM CM for 12 h were measured by qRT-PCR, n = 3. (I-K) The anti-tumor activity and TME remodeling by 4-OI-treated DCs in C57BL/6 mice, n = 5. The tumor growth curve (I), tumor image (J), and tumor weight (K) of B16-F10-OVA-beared mice after administration with DCs or 4-OI-treated DCs subcutaneously at day 5. (L) The proportion of immune cells and CD3^+^, CD4^+^, and CD8^+^ T cells within TME after treatment with DCs or 4-OI-treated DCs compared to the control group. (M) The percentage of IFNγ^+^ CD4^+^ T cells among CD4^+^T cells in the TME. (N) The statistics and presentative data of IFNγ^+^ CD8^+^ T cells in the TME. ns = no significance, * p < 0.05, ** p < 0.01, *** p < 0.001.

*Plasmodium*-induced itaconate restrains monocyte-derived DCs by disrupting the mitochondria, leading to the release of nucleic acids, which induces PD-L1 expression in DCs, and then impairs CD8^+^ T cell activation 36. DCs are critical APCs after mRNA-LNP stimulation and are activated to present antigens, express co-stimulatory molecules, and transition from innate to adaptive immunity 33. Thus, we were curious to determine whether itaconate affects the function of DCs after OVA-LNP treatment. We detected OVA and *Irg1* expression in BMDMs and BMDCs after stimulation with OVA-LNP for 12 h. The results indicated that OVA expression was significantly higher upregulated in BMDMs than in BMDCs (**Figure 4A**), and *Irg1* expression was markedly increased in BMDMs after OVA-LNP treatment, with no significant change observed in BMDCs (**Figure 4B**). Itaconate secretion was detected in the supernatant of both BMDMs and BMDCs at 24 h using liquid chromatography-mass spectrometry (LC/MS) analysis. We observed a much more significant increase of itaconate in BMDMs rather than in BMDCs, consistent with *Irg1* expression (**Figure 4C**). These findings suggest that OVA-LNP-induced *Irg1* and itaconate are primarily derived from macrophages, rather than DCs. BMDMs from WT and *Irg1*^-/-^ mice were used to collect conditioned medium (CM) after OVA-LNP treatment for 24 h, and then BMDCs were cultured using this CM (**Figure S5A**). We measured itaconate in the supernatants of WT and *Irg1*^-/-^ BMDMs and observed high itaconate levels secreted in OVA-LNP-treated WT BMDMs but not in *Irg1*^-/-^ BMDMs (**Figure 4D**). After culturing in the CM for 24 h, we found that CM derived from *Irg1*-depleted BMDMs upregulated CD80, CD86, and MHC II of BMDCs compared to WT BMDMs (**Figure 4E-G**,** S5B**). In addition, *Il1β, Il8, Cxcl9, Cxcl10,* and *Ccr7* mRNA expression were measured, and were considerably higher in BMDCs treated with *Irg1*-depleted BMDMs than WT BMDMs (**Figure 4H**). We next treated BMDCs with 4-OI *in vitro*. The results demonstrated that while 4-OI did not affect BMDC viability, it significantly suppressed their proliferation (Figure S5C-D). To rule out the influence of pro-inflammatory cytokines released by BMDMs, BMDCs were supplemented with 4-OI following Irg1^-/-^-CM treatment. This approach revealed that 4-OI treatment reduced the expression of OVA, MHC I, and MHC II in BMDCs (**Figure S5E-G**).

To confirm the role of itaconate in the function of DCs* in vivo*, we isolated BMDCs and exposed them to OVA peptide and 4-OI. We then transferred these BMDCs to mice bearing B16-F10-OVA tumor cells at day 5. The injection of DCs significantly reduced tumor growth compared to that in the control group, and this effect was inhibited by 4-OI (**Figure 4I-K**). Within the TME, the transfer of DCs led to a significant increase in immune cells and CD3^+^, CD4^+^, and CD8^+^ T cells (**Figure 4L**). This increase was accompanied by an increase in IFNγ^+^ CD4^+^ and IFNγ^+^ CD8^+^ T cells (**Figure 4M-N**). Furthermore, 4-OI diminished the activation of the TME by DCs (**Figure 4L-N**). These results indicate that itaconate produced by macrophages in response to OVA-LNP inhibits DC function.

### Encapsulating si-*Irg1* with OVA mRNA in LNP efficiently decreases itaconate and enhances APCs and T cell function *in vitro* and *in vivo*

**Figure 5:**
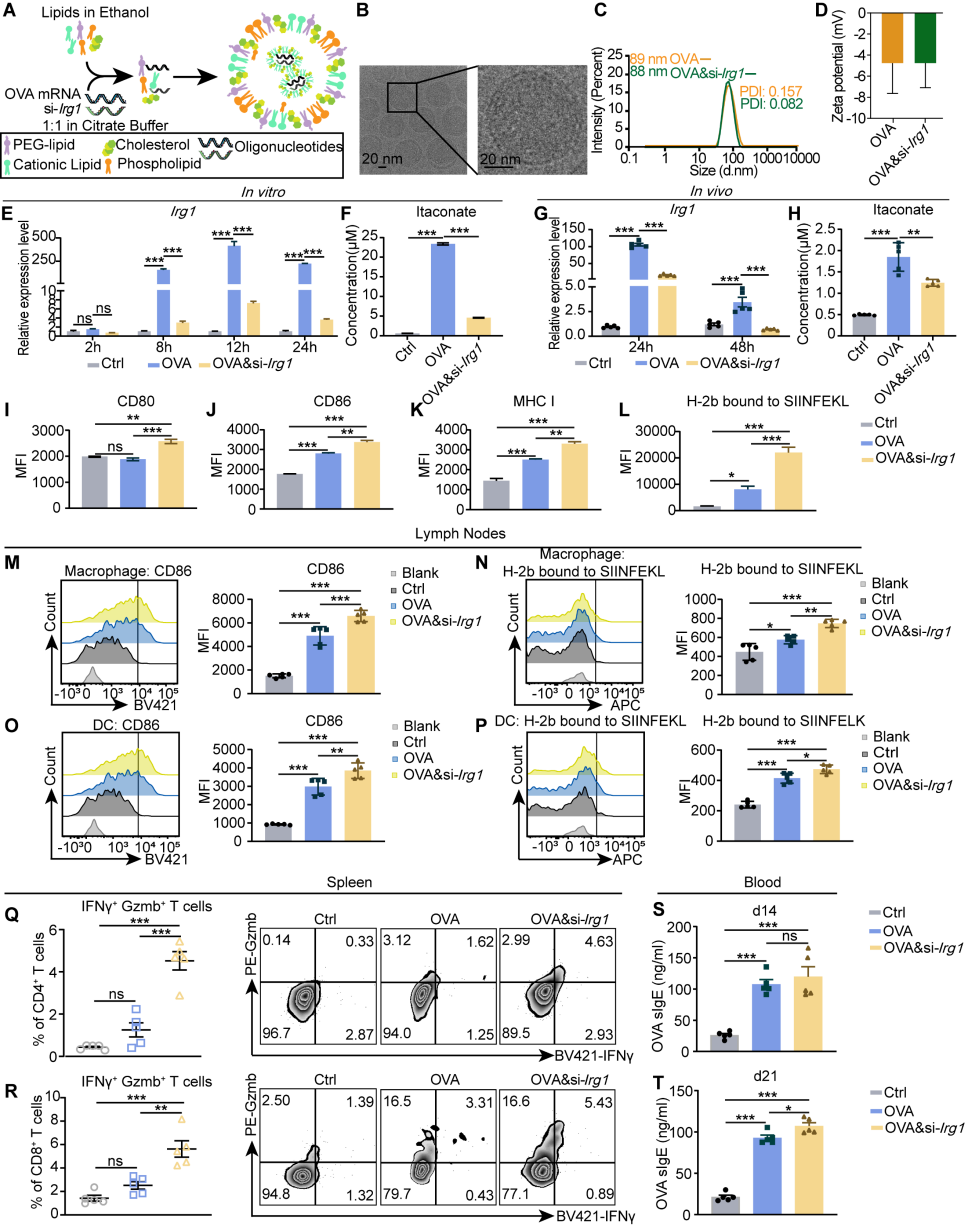
Targeting *Irg1* on macrophages in LNs. (A) The diagram of the process of encapsulating OVA&si-*Irg1*-LNP. (B) The structure of OVA&si-*Irg1*-LNP by cryo-TEM. (C-D) The diameter (C) and zeta potential (D) of OVA-LNP and OVA&si-*Irg1*-LNP. (E) *Irg1* expression levels of BMDM after treatment with 0.3 μg/mL OVA-LNP and OVA&si-*Irg1*-LNP for different time points detected by qRT-PCR, n = 3. (F) The itaconate concentration of supernatant of BMDM after treatment with 0.3 μg/mL OVA-LNP and OVA&si-*Irg1*-LNP for 24 h was detected by LC/MS, n = 3. (G) *Irg1* expression levels of LNs after treatment with 5 μg OVA-LNP and OVA&si-*Irg1*-LNP subcutaneously for 24 and 48 h were detected by qRT-PCR, n = 5. (H) The itaconate concentration of intertissue fluid of LNs after treatment with 5 μg OVA-LNP and OVA&si-*Irg1*-LNP for 24 h was detected by LC/MS. (I-L) CD80 (I), CD86 (J), MHC I (K), and H-2^b^ bound to SIINFEKL (L) of BMDM after treatment with 0.3 μg/mL OVA-LNP and OVA&si-*Irg1*-LNP for 24 h were detected by flow cytometry, n = 3. (M-P) The CD86 and H-2^b^ bound to SIINFEKL of macrophages (M and N) and DC (O-P) in LNs after treatment with 5 μg OVA-LNP and OVA&si-*Irg1*-LNP subcutaneously in C57BL/6 mice for 24 h were detected by flow cytometry, n = 5. (Q-R) The statistic and presentative data of IFNγ^+^ Gzmb^+^ CD4^+^(Q), IFNγ^+^ Gzmb^+^ CD8^+^ (R) T cells in spleens were detected by flow cytometry after treatment with 5 μg OVA-LNP and OVA&si-*Irg1*-LNP subcutaneously in C57BL/6 mice for 21 days, n = 5. (S-T) The concentration of OVA sIgE in blood serum at days 14 (S) and 21 (T) was detected by ELISA with 5 μg OVA-LNP and OVA&si-*Irg1*-LNP injection subcutaneously, n = 5. ns = no significance, * p < 0.05, ** p < 0.01, *** p < 0.001.

As mentioned, itaconate inhibits tumor suppression and reduces the immune response to mRNA cancer vaccines. To reduce the production of itaconate from macrophages, we synthesized si-*Irg1* siRNA and encapsulated it along with OVA mRNA into the same LNP (OVA&si-*Irg1*-LNP) to deliver exogenous mRNA and siRNA to macrophages in the LNs (**Figure 5A**). The OVA&si-*Irg1*-LNP exhibited a uniform spherical shape with a “membrane-core” structure, as observed by cryo-transmission electron microscopy (cryo-TEM) (**Figure 5B**). We characterized the OVA-LNP and OVA&si-*Irg1*-LNP formulations, measuring particle sizes of approximately 80-100 nm using dynamic light scattering (DLS) (**Figure 5C**). The zeta potentials of these LNPs ranged from -15 to +15 (**Figure 5D**). Both formulations exhibited high encapsulation efficiency (≥ 90%) with mRNA concentrations of ≥ 0.100 mg/mL (Figure S6A). To serve as a control for si-*Irg1*, we designed si-Ctrl and encapsulated it in OVA&si-Ctrl-LNP. No significant differences were observed between OVA-LNP and OVA&si-Ctrl-LNP in promoting *Irg1* and itaconate upregulation (**Figure S6B-C**). Additionally, LNP stimulation had no noticeable impact on BMDM viability or proliferation (**Figure S6D-E**). In this study, we assessed the effect of siRNAs on BMDMs. Our findings revealed that *Irg1* expression was significantly reduced with OVA&si-*Irg1*-LNP compared to that with OVA-LNP after 8 h, whereas the expression of OVA was unaffected (**Figure 5E**, **S6F**). Additionally, itaconate levels in the supernatant were much lower in OVA&si-*Irg1*-LNP than in OVA-LNP (**Figure 5F**). Additionally, we measured OVA and *Irg1* expression in the iLNs after subcutaneous injection. Our findings indicated that OVA&si-*Irg1*-LNP effectively downregulated *Irg1* without affecting OVA expression at 24 and 48 h (**Figure 5G**, **S6G**). Furthermore, itaconate levels in the LNs were reduced following OVA&si-*Irg1*-LNP injection (**Figure 5H**). We also measured the expression level of OVA and *Irg1* in the spleen. The data revealed that OVA was upregulated in both groups at 24 and 48h, whereas *Irg1* was downregulated at 48 h. However, the expression levels of both OVA and *Irg1* were considerably lower than those in the LNs (**Figure S6H-I**). Therefore, the reduction of itaconate levels using OVA&si-*Irg1*-LNP is a promising strategy for improving the efficacy of cancer vaccines.

Next, we certified its effects on APCs and T cells. We isolated BMDMs, treated them with OVA-LNP and OVA&si-*Irg1*-LNP, and determined their activation states using flow cytometry. The data showed that OVA&si-*Irg1*-LNP upregulated CD80, CD86, MHC I, and H-2^b^ bound to SIINFEKL compared with OVA-LNP (**Figure 5I-L**). Next, LNPs were injected subcutaneously into mice, iLNs at 24 h, spleens on day 21, and blood serum on days 14 and 21 were collected. The levels of CD86 and H-2^b^ bound to SIINFEKL of macrophages and DCs in the iLNs were upregulated in the OVA-LNP group compared to those in the control group. Moreover, these levels were higher in the OVA&si-*Irg1*-LNP group in both macrophages (**Figure 5M-N**) and DCs (**Figure 5O-P**). Spleen cells were obtained and pulsed with OVA peptide for 16 h, followed by monensin treatment for 4 h. IFNγ and granzyme B (Gzmb) levels were detected by flow cytometry. The data showed that OVA&si-*Irg1*-LNP increased IFNγ^+^ Gzmb^+^ T cells of CD4^+^ and CD8^+^ T cells compared to OVA-LNP (**Figure 5Q-R**). In addition, the OVA sIgE antibody levels increased on days 14 and 21 in both groups compared to those in the control group, with significantly higher levels observed in the OVA&si-*Irg1*-LNP group on day 21 (**Figure 5S-T**). These data demonstrate that targeting *Irg1* with LNPs efficiently enhances the function of APCs and T cells *in vitro* and *in vivo*.

### Treatment with OVA&si-*Irg1*-LNP promotes therapeutic efficacy and remodels the TME in the B16-F10 melanoma mouse model

**Figure 6:**
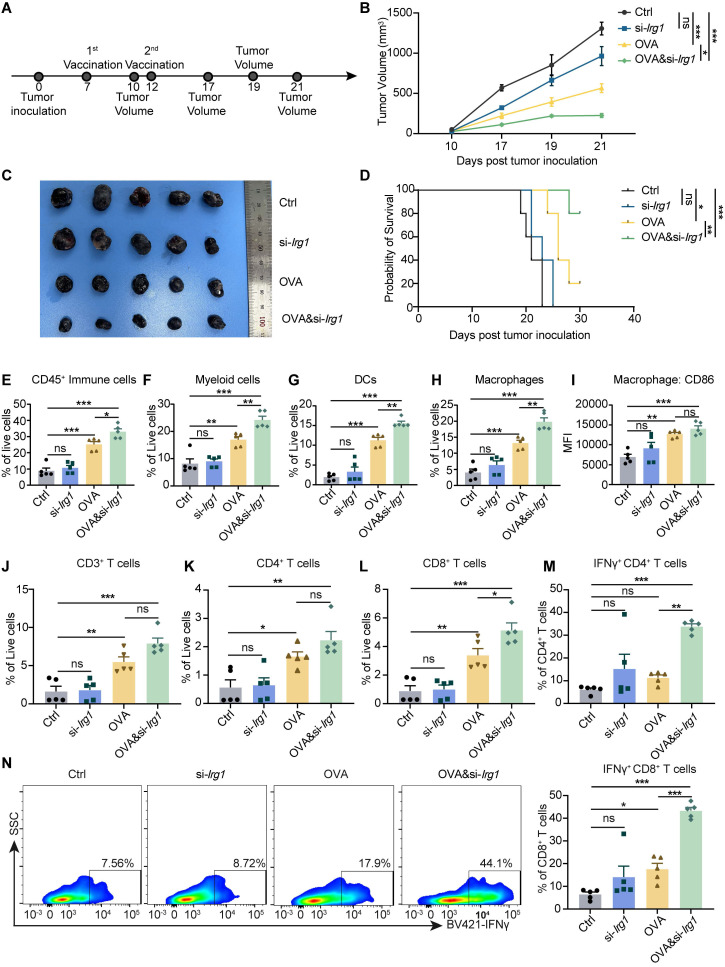
OVA&si-*Irg1*-LNP promoted therapeutic efficacy and remodeled the tumor microenvironment in the B16-F10 melanoma mouse model. (A)The schematic diagram of the B16-F10-OVA-beared melanoma mouse model, n = 5. (B-C) The tumor growth curve (B) and tumor image (C) of the B16-F10-beared mice after administration with two-dose LNPs. (D) Kaplan-Meier analysis of B16-F10-OVA melanoma mice. (E-N) The percentage of immune cells in the TME was detected by flow cytometry after LNP treatment for 21 days. (E-I) The proportion of CD45^+^ immune cells (E), CD11b^+^ myeloid cells (F), CD11c^+^ DCs (G), F4/80^+^ macrophages (H), CD86 expression level of macrophages (I) within TME. (J-N) Flow cytometry analysis of CD3^+^ (J), CD4^+^ (K), CD8^+^ (L), IFNγ^+^ CD4^+^ (M) T cells, and the presentative and statistic proportion of IFNγ^+^ CD8^+^ T cells (N) in the TME after LNPs treatment. ns = no significance, * p < 0.05, ** p < 0.01, *** p < 0.001.

mRNA cancer vaccines have shown great promise in preclinical studies and have been tested in clinical trials. OVA&si-*Irg1*-LNP have been demonstrated to enhance APC and T cell functions. To confirm the anti-tumor therapeutic effect, we established a B16-F10-OVA tumor model, and 5 μg OVA-LNP, si-*Irg1*-LNP, or OVA&si-*Irg1*-LNP was administered subcutaneously on day 7 when the tumor volume reached 50mm^3^, and a boost dose was given on day 12 (**Figure 6A**). Control mice displayed rapid tumor growth, which was slightly inhibited by si-*Irg1*-LNP. However, OVA-LNP significantly suppressed tumor growth, and OVA&si-*Irg1*-LNP inhibited tumor growth more effectively than OVA-LNP (**Figure 6B-C**). The survival rate was consistent with tumor growth (**Figure 6D**).

As previously mentioned, OVA&si-*Irg1*-LNP significantly enhanced the activation and antigen presentation of macrophages and DCs in the iLNs, as well as the anti-tumor functions of T cells in the spleen. At the end of the therapeutic tumor model, we evaluated the infiltration of macrophages, DCs, and T cells within the TME using flow cytometry (**Figure S7A-B**). The results indicated that OVA&si-*Irg1*-LNP significantly increased the number of immune cells, myeloid cells, DCs, and macrophages (**Figure 6E-H**) within the TME compared to OVA-LNP. Additionally, CD86 expression in macrophages was slightly but not significantly upregulated (**Figure 6I**). T cells play a critical role in anti-tumor efficacy, and we found that the proportion of CD3^+^ and CD4^+^ T cells was slightly increased in the OVA&si-*Irg1*-LNP group compared to that in the OVA-LNP group (**Figure 6J-K**). However, CD8^+^, IFNγ^+^ CD4^+^, and IFNγ^+^ CD8^+^ T cells were significantly increased by OVA&si-*Irg1*-LNP (**Figure 6L-N**). We administered LNPs at two doses, and one month later, established the B16-F10 tumor mouse model to evaluate their protective efficacy (**Figure S7C**). The results demonstrated that OVA&si-*Irg1*-LNP was more effective in inhibiting tumor growth and prolonging mouse survival compared to controls (**Figure S7D-E**). Body weight was monitored after a single subcutaneous injection to investigate the safety of OVA-LNP, si-*Irg1*-LNP, and OVA&si-*Irg1*-LNP *in vivo*. Body weights were unchanged compared with those in the control group (**Figure S8A**). Alanine transaminase (ALT) and aspartate transaminase (AST), used in liver function tests, and blood urea nitrogen (BUN) and creatinine (CR), used in kidney function tests, were measured using biochemical assays on days 7, 14, and 21. All these markers were below normal levels and with no obvious changes in the four groups (**Figure S8B-E**). After vaccination, the heart, liver, spleen, lung, and kidney tissues were collected, and hematoxylin and eosin (H&E) staining was performed. The results showed no apparent changes in the main organs, indicating that the LNPs were safe *in vivo* (**Figure S8F**).

In summary, OVA&si-*Irg1*-LNP treatment induced enhanced anti-tumor effects, increased myeloid and T cell infiltration into the TME, and demonstrated good safety. These data indicate that targeting *Irg1* has excellent potential for tumor therapy.

### Targeting itaconate enhances the anti-tumor activity of cancer vaccine in combination with anti-PD-1 antibody *in vivo*

**Figure 7:**
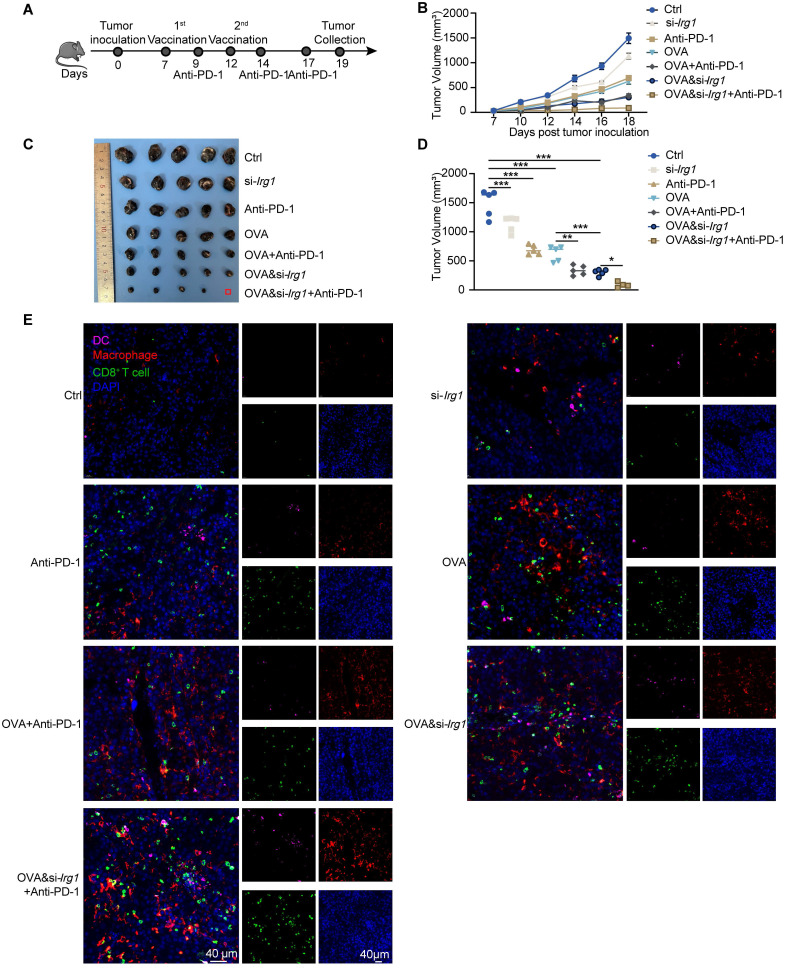
OVA&si-*Irg1*-LNP enhanced the anti-tumor efficacy of anti-PD-1 antibody. (A) The schematic diagram showed the administration of LNPs alone or combined with an anti-PD-1 antibody in a B16-F10-OVA-bearing melanoma mouse model, n = 5. (B-D) The tumor growth (B), tumor image (C), and tumor volume at day 19 (D) of the B16-F10-OVA-beared mice. (E) The immune cells, like DCs (CD11b), macrophage (F4/80), and CD8^+^ T cells (CD8), were detected by immunofluorescence in the TME. ns = no significance, * p < 0.05, ** p < 0.01, *** p < 0.001.

Combining cancer vaccines with anti-PD-1 antibodies is an effective strategy for improving the therapeutic effects against cancer. We injected B16-F10-OVA cells subcutaneously on day 0, administered vaccines on days 7 and 12, and anti-PD-1 antibodies on days 9, 14, and 17 (**Figure 7A**). We observed that a combined therapeutic strategy enhanced the anti-tumor activity of the anti-PD-1 antibody. Combining OVA&si-*Irg1*-LNP with anti-PD-1 antibodies yielded the most effective therapeutic results (**Figure 7B-D**). Immune cells, such as DCs, macrophages, and CD8^+^ T cells, were measured using immunofluorescence. The data revealed that both LNPs and anti-PD-1 antibodies, either alone or in combination, increased the infiltration of immune cells compared with the untreated group. In the combined treatment groups, a higher proportion of macrophages and CD8^+^ T cells were present within the TME compared to treatment with LNPs alone. Importantly, OVA&si-*Irg1*-LNP combined with the anti-PD-1 antibody led to significantly more DC infiltration into the TME than the other treatments (**Figure 7E**). These results demonstrated that combining a cancer vaccine with an anti-PD-1 antibody improved anti-tumor efficacy, and targeting *Irg1* enhanced the combined therapy.

## Discussion

mRNA possesses intrinsic adjuvant activity and can be recognized by specific PRRs of the innate immune system, boosting the full activation of adaptive immunity. The immune system displays potent immunostimulatory capabilities, activating immune responses while simultaneously initiating negative feedback mechanisms to prevent excessive or prolonged activation. This regulation involves various pathways, including glucocorticoids, which not only induce an inflammatory state but also trigger a systemic increase in itaconate, contributing to its anti-inflammatory effects 37. Moreover, D-2-hydroxyglutarate exhibits anti-inflammatory effects and has been shown to accumulate in macrophages following TLR activation 38. IFNγ, primarily produced by CD8⁺ cytotoxic T lymphocytes, plays a critical role in antitumor immunity. However, studies have shown that IFNγ can also upregulate PD-L1 expression on tumor cells, thereby facilitating tumor immune evasion and promoting tumor progression 39-42. In this study, we observed significant changes in metabolites following stimulation with an mRNA cancer vaccine, with itaconate being the most upregulated metabolite. Itaconate suppressed antigen presentation by DCs and decreased the efficacy of mRNA cancer vaccines. These data demonstrated that changes in the metabolic environment of LNs play a critical role in the immune response to cancer vaccines. Recent studies have shown that exposure to a high-fat diet limits T cell maturation in the memory compartment during influenza vaccination, corresponding to changes in systemic obesity-related biomarkers such as leptin and adiponectin 43. patients with melanoma who received DC vaccines showed that clinical outcomes correlate with the DC metabolic profile, and that metabolism is related to the immune phenotype 44. These studies suggest that alterations in metabolite profiles are correlated with vaccine efficacy and that targeting abnormal metabolites may improve the immune response to vaccines. However, alterations in metabolites and their roles in mRNA cancer vaccines have not yet been studied.

Itaconate binds to diverse proteins intracellularly, regulating metabolism, oxidative responses, epigenetic modifications, and gene expression, while also signaling extracellularly through G protein-coupled receptors 25, 45. Itaconate is an anti-inflammatory metabolite in inflammatory diseases, but it also promotes tumorigenesis and inhibits anti-tumor immunotherapy by impairing immune cell function 46, 47. Recent studies have identified SLC13A3 as an importer of itaconate, with SLC13A3-mediated uptake contributing to tumor immune evasion, resistance to ferroptosis, and hepatic antibacterial innate immunity 48-50. In this study, we found that macrophage-derived itaconate suppresses the activation and antigen-presentation of DCs in iLNs. However, whether itaconate induced by mRNA-LNPs in iLNs is taken up by DCs remains to be further investigated. The same metabolite can have opposing effects on tumor cells and immune cells. Itaconate, produced by polymorphonuclear myeloid-derived suppressor cells (PMN-MDSCs), myeloid cells such as monocytes and macrophages, or tumor-infiltrating neutrophils, promotes tumor progression by suppressing CD8^+^ T cell proliferation and function 30, 31. However, a recent study revealed that tumor-intrinsic itaconate production can enhance CD8^+^ T cell infiltration and activation by modulating tumor immunogenicity 51. These findings suggest that the same metabolite may exert divergent effects—suppressive or stimulatory—depending on whether it acts intracellularly within tumor cells or extracellularly on neighboring immune cells. In this study, we demonstrated that OVA-LNP-induced itaconate, which is derived from macrophages in iLNs, reduces the immune response to cancer vaccines by impairing antigen presentation and the activation of DCs and inhibiting the anti-tumor function of T cells. Targeting itaconate with si-*Irg1* effectively reduced itaconate levels in iLNs and improved the anti-tumor efficacy of the mRNA cancer vaccine. Currently, *Irg1* depletion in mice has been used to target itaconate in cancer immunotherapy 30, 32, 52-54. However, exogenous depletion has not been investigated. We propose a new strategy for targeting itaconate in LNs, which enhances the effectiveness of cancer vaccines with good safety. This may provide a new strategy for improving the efficacy of mRNA cancer vaccines.

Our study focused on itaconate induction by mRNA cancer vaccines and its inhibitory functions, and we proposed a strategy to enhance tumor suppression by mRNA cancer vaccines. In the future, it will be important to investigate whether itaconate production depends on the type of vaccine used, such as peptide- or DNA-based vaccines. We used OVA as a model antigen, which is specifically expressed in stably transfected B16-F10-OVA cell lines. Further research is required to investigate the role of itaconate in other tumor-associated and tumor-specific antigens (like HER2, KRAS mutations). Nonetheless, identifying itaconate and its immunosuppressive biological functions in mRNA cancer vaccines, and providing a strategy to inhibit its inhibitory functions offer the possibility of enhancing the effectiveness of cancer immunotherapies.

## Conclusion

Our results indicate that itaconate is the most upregulated metabolite in activated iLNs compared to that in homeostatic cLNs after OVA-LNP stimulation. Depletion of *Irg1* improved T-cell mediated tumor killing and antibody production by B cells. We demonstrated that itaconate induced by OVA-LNP was mainly derived from macrophages in the LNs, which is consistent with previous tumor studies 30, 54, 55. We observed an enhanced pro-inflammatory state in macrophages after *Irg1* depletion and demonstrated that OVA-LNP induced *Irg1* expression *via* multiple PPR pathways. We also demonstrated that DCs are the target cells of macrophage-derived itaconate in the LNs. To enhance the efficacy of mRNA cancer vaccines, we designed OVA&si-*Irg1*-LNP, which encapsulated OVA and si-*Irg1* into the same LNP. The expression of *Irg1* and itaconate was effectively downregulated in BMDMs and LNs, leading to an enhanced pro-inflammatory state in BMDM and heightened macrophage, DC, and T cell responses to cancer vaccines. Importantly, OVA&si-*Irg1*-LNP demonstrated potent anti-tumor efficacy when used alone or in combination with an anti-PD-1 antibody and also increased the infiltration of immune cells, including DCs, macrophages, and T cells. In addition, we observed good safety of OVA&si-*Irg1*-LNP. We found that macrophage-derived itaconate impaired the efficacy of cancer vaccines, and demonstrated a method to circumvent this activity, which may provide a strategy to improve the therapeutic efficacy of mRNA cancer vaccines.

## Supplementary Material

Supplementary figures and tables.
