# Supplementary material for: Boosting mRNA cancer vaccine efficacy via targeting Irg1 on macrophages in lymph nodes
Source: Theranostics. 2025 May 25;15(13):6329–46. doi: 10.7150/thno.110305 (PMC12159843; doi:10.7150/thno.110305)
Supplement: Supplementary file 1 — Supplementary figures and tables. [file thnov15p6329s1.pdf]

Boosting mRNA cancer vaccine efficacy via targeting *Irg1* on macrophages in lymph nodes

Wenwen Wei[#1,2,3], Xiao Yang[#1,2,3], Yeshan Chen[#1,2,3], Mengjie Che[#1,2,3], Ying Ye[1,2,3], Yue Deng[1,2,3], Mengyao Su[1,2,3], Yajie Sun[1,2,3], Jingshu Meng[1,2,3], Yan Hu[1,2,3], Jiacheng Wang[1,2,3], Yijun Wang[1,2,3], Zishan Feng[1,2,3], Zhiyuan Zhou[1,2,3], Yan Li[1,2,3], Qian Li[1,2,3], Zhanjie Zhang[1,2,3],Bian Wu[1,2,3], Haibo Zhang[4], You Qin[1,2,3], Lu Wen[1,2,3], Chao Wan*[1,2,3], Kunyu Yang*[1,2,3]

1. Cancer Center, Union Hospital, Tongji Medical College, Huazhong University of Science and Technology, Wuhan 430022, China

2. Institute of Radiation Oncology, Union Hospital, Tongji Medical College, Huazhong University of Science and Technology, Wuhan 430022, China

3. Hubei Key Laboratory of Precision Radiation Oncology, Wuhan 430022, China

4. Cancer Center, Department of Radiation Oncology, Zhejiang Provincial People's Hospital (Affiliated People's Hospital), Hangzhou Medical College, Hangzhou, Zhejiang 310000, China

[#] These authors have contributed equally to this article.

* Corresponding authors: E-mail: Kunyu Yang, yangkunyu@hust.edu.cn; Chao Wan, wanc@hust.edu.cn

The PDF file includes:

Supplementary Materials and Methods

Figs. S1 to S8

Tables S1 to S2

Supplementary Materials and Methods

Single-cell RNA sequencing

The single cells were loaded into the microfluidic chip of Chip A Single Cell Kit v2.1 (S050100301) to generate droplets with MobiNova-100 (A1A40001). Each cell was encapsulated in a droplet containing a gel bead linked with millions of unique oligos (cell barcodes). After encapsulation, droplets were cut with light by MobiNovaSP-100 (A2A40001) while oligos diffused into the reaction mix. A unique cell barcode labeled mRNA with cDNA amplification in droplets. Following cDNA amplification with barcode, a library was constructed using the High Throughput Single-Cell 3' Transcriptome Kit v2.1 (S050200301) and the 3' Dual Index Kit (S050300301). The libraries were then sequenced on an Illumina NovaSeq 6000 System.

The FASTQ files were initially processed and aligned to the Mus musculus reference GRCm39 using MobiVision software (version 2.1). Unique molecular identifier (UMI) counts were then aggregated for each barcode, and the count matrix was analyzed using the Seurat R package (version 4.0.0). We filtered out poor-quality cells and potential multiple captures based on the following criteria: (1) gene numbers < 200, (2) UMI < 1000, (3) log10GenesPerUMI < 0.7, (4) proportion of UMIs mapped to mitochondrial genes > 10%, and (5) proportion of UMIs mapped to hemoglobin genes > 5%. Subsequently, we used the DoubletFinder package (version 2.0.3) to identify potential doublets and multiplets and the NormalizeData function to normalize

library size. Specifically, the gene expression measurements for each cell were normalized using the global-scaling normalization method "LogNormalize", which involved multiplying the total expression by a scaling factor (default is 10,000) and then log-transforming the results.

The cells were clustered based on their gene expression using the FindClusters function. A 2-dimensional Uniform Manifold Approximation and Projection (UMAP) algorithm was used for visualization with the RunUMAP function. Marker genes for each cluster were identified using the FindAllMarkers function (test. use = presto). Differentially expressed genes (DEGs) were selected using the FindMarkers function (test. use = presto), with a significance threshold of P value < 0.05 and $|log2foldchange| > 0.58$. GO enrichment and KEGG pathway enrichment analyses of DEGs were conducted using R (version 4.0.3) based on the hypergeometric distribution.

***In vitro* tumor killing assay**

After injecting OVA-LNP, single cells were isolated from the spleens of both WT and *Irg1*$^{-/-}$ mice on day 21. Next, spleen cells were cocultured with B16-F10-OVA cells at an E: T ratio of 25: 1. After 24 h, the cells were collected and stained with 7-AAD (420404, Biolegend) to label dead cells. The cytotoxicity activity was then measured using flow cytometry.

Inhibitors

The PRRs inhibitor MYD88 inhibitor (HY-149992, MedChemExpress), NOD1 inhibitor (HY-100691, MedChemExpress), and RIG1 inhibitor (HY-147124, MedChemExpress) were dissolved in DMSO. 10 μM MYD88i, 10 μM NOD1i, and 500

nM RIG1i combined with OVA-LNP were used to treat BMDMs.

Western blotting (WB) analysis

BMDMs were treated with 0.3 μg/mL OVA-LNP and 10 μM MYD88i, 10 μM NOD1i, and 500 nM RIG1i for 24 h. The cells were lysed with RIPA lysis buffer (G2002, Servicebio) with 1% protease inhibitors (G2008, Servicebio) and 1% phosphatase inhibitors (G2007, Servicebio) for 30 min. The supernatant was collected and boiled with 5×loading buffer. The samples were resolved by sodium dodecyl sulfate-polyacrylamide gel electrophoresis (SDS-PAGE) and then transferred to polyvinylidene fluoride (PVDF) membranes. Then, the membranes were blocked with 5 % non-fat milk at RT for 1 h, incubated with primary antibodies at 4 ℃ overnight, and secondary antibodies at RT for 1 h. The signals were obtained using ECL reagents (G2020, Servicebio) in the dark room. The primary antibodies used in this study were: GAPDH (60004-1-Ig, Proteintech, 1:5000); IRF3 (66670-1-Ig, Proteintech, 1:2000); Phospho-IRF3 (29528-1-AP, Proteintech, 1:1000); c-JUN (66313-1-Ig, Proteintech, 1:2000); Phospho-c-JUN (80086-1-RR, Proteintech, 1:1000); NF-κB p65 (10745-1-AP, Proteintech, 1:1000); Phospho-NF-κB p65 (82335-1-AP, Proteintech, 1:1000); IRG1 (ab222411, Abcam, 1:500).

LC/MS analysis

The 200 μL of supernatant from BMDM or BMDC was added with 600 μL of protein precipitant methanol-ethylene solution (V: V=2: 1). The mixture was vortexed for 3 min and incubated at -20 °C for 30 min. After incubation, the samples were centrifuged for 10 min at 13000 rpm at 4 °C, and 600 μL of supernatant was transferred

to the injection vial. This was followed by another incubation at -20 °C for 30 min and centrifugation at 12000 rpm for 3 min at 4 °C. Finally, 400 μL of supernatant was transferred to the liner of the injection vial for LC/MS analysis.

For intertissue fluid, the whole LNs and 30 mg liver, spleen, and injection site were collected 24 h after a subcutaneous injection of 5 μg LNPs and transferred into a 600 μL methanol-water solution (V: V=4: 1). The samples were then homogenized with pre-cooled beads using an ultrasonic homogenizer at 60 Hz for 2 min, followed by vortexing for 5 min. After that, they were incubated at -20 °C for 30 min. Subsequently, the solution was centrifuged at 13000 rpm for 10 min at 4 °C. 500 μL of the supernatant was transferred into another tube and incubated at -20 °C for 30 min. Finally, centrifuge the solution at 12000 rpm for 3 min at 4 °C and transfer 400 μL of the supernatant into the injection vial for LC/MS analysis.

Adoptive DCs transfer for tumor therapy

The bone marrow-derived cells from female C57BL/6 mice, aged 6-10 weeks, were collected and then differentiated in RPMI 1640 with 10% FBS and 20 ng/mL granulocyte-macrophage colony-stimulating factor (GM-CSF) (576306, biolegend) to obtain BMDCs. Six days later, the BMDCs were stimulated with 0.3 μg/mL OVA-LNP or 125 μM 4-OI for 12 h. After stimulation, the BMDCs were washed and suspended in PBS. Then, they were transferred (1×10^6 per mouse) subcutaneously adjacent to the tumors on day 5 after the B16-F10-OVA injection.

Macrophage depletion

Clodronate liposomes (FormuMax, F70101C-AC) were administered intraperitoneally

to mice at a dose of 200 μL. Peripheral blood was collected to detect depletion efficacy. After 24 h, each mouse received a subcutaneous injection of 5 μg OVA-LNP. Tissue samples, including the liver, spleen, iLN, cLN, and blood serum, were harvested 24 hours post-OVA-LNP injection for the detection of *Irg1* and itaconate.

Enzyme-linked immunosorbent assay

Blood was collected from Orbital venous plexus WT and $Irg1^{-/-}$ mice. Then, the blood was incubated at RT for 20 min and centrifuged at 2000 g for 15 min to obtain the serum. The OVA-specific sIgE of blood serum was measured with an ELISA kit (EM2035, FineTest) according to the manufacturer's protocol.

Immunofluorescence

To visualize mouse immune cells in LNs and TME, the LNs and tumor samples were fixed in 4% paraformaldehyde, and 4 μm paraffin-embedded sections were prepared. The sections underwent deparaffinization and hydration, followed by treatment with 3% H_2O_2 solution for 20 min to sequester peroxidase. To retrieve antigenicity, the sections were boiled with sodium citrate antigen repair solution in a microwave: 5 min in high heat, 8 min in medium heat, and 10 min in low heat. Following this, the sections were blocked in donkey serum at room temperature (RT) for 1 h. They were then stained with primary antibodies for CD3, CD19, F4/80, CD11c, and CD8 at 4 °C overnight, followed by secondary antibodies at RT for 1 h. Finally, the sections were counterstained with DAPI, and images were captured with a fluorescence microscope.

Cry-transmission electron microscopy

The Cu girds (300 mesh, 1.2/1.3) were treated, and the Au grids (300 mesh, 1.2/1.3 Ni-Ti) were treated at 15 mA for 50s using PELCO easiGlow Discharge (TED PELLA). Subsequently, 3 μL LNPs were dropped on the discharged grid (bolt time 4 s, bolt force 0, wait time 30 s) using Vitrobot™ Mark IV (MARK IV, Thermo Fisher) to prepare frozen samples. After assembling the frozen grid and plunge-freezing it under liquid nitrogen to form a complete cartridge, the completed cartridge should be placed into the cassette and loaded into the autoloader. Images were captured on Glacios™ 2 Cryo-TEM (GLACIOSTEM, Thermo Fisher).

Supplementary Figures

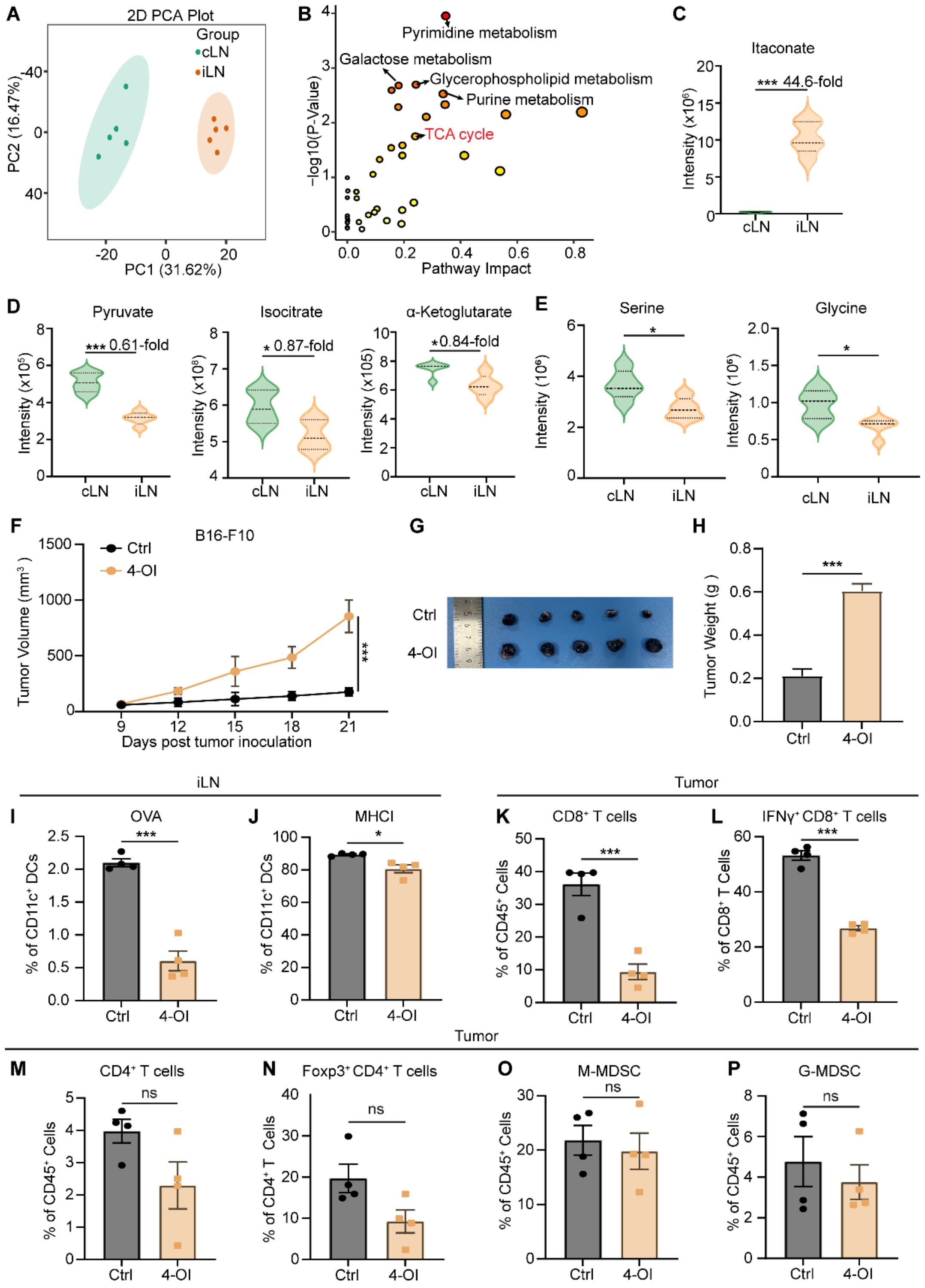

Figure S1. OVA-LNPs induce itaconate in iLNs inhibits anti-tumor efficiency.

(A-E) Analysis of the non-targeted profile of water-soluble metabolites. (A) The PCA of metabolites of iLNs and cLNs. PC, principal component. (B) The metabolite

pathways enriched in iLNs compared to cLNs. (C-E) The levels of itaconate, pyruvate, isocitrate, α-ketoglutarate, serine and glycine in iLNs and cLNs with OVA-LNP stimulation. (F-P) The tumor growth and TME of *Irg1*$^{-/-}$ mice with OVA-LNP at days 7 and 12, and 4-OI (50mg/kg) at days 7-14 for every day, n = 4. (F-H) Tumor growth curve (F), tumor image on day 21 (G), and tumor weight (H). (I-J) The OVA (I) and MHC I (J) of DCs in iLNs. (K-N) The CD8$^+$ (K), IFNγ$^+$ CD8$^+$ (L), CD4$^+$ (M), and Foxp3$^+$CD4$^+$ (N) T cells, and M-MDSC (CD11b$^+$Ly6C$^+$Ly6G$^-$, O), G-MDSC (CD11b$^+$Ly6C$^-$Ly6G$^+$, P) within the TME between Ctrl and 4-OI groups. ns = no significance, * $p < 0.05$, ** $p < 0.01$, *** $p < 0.001$.

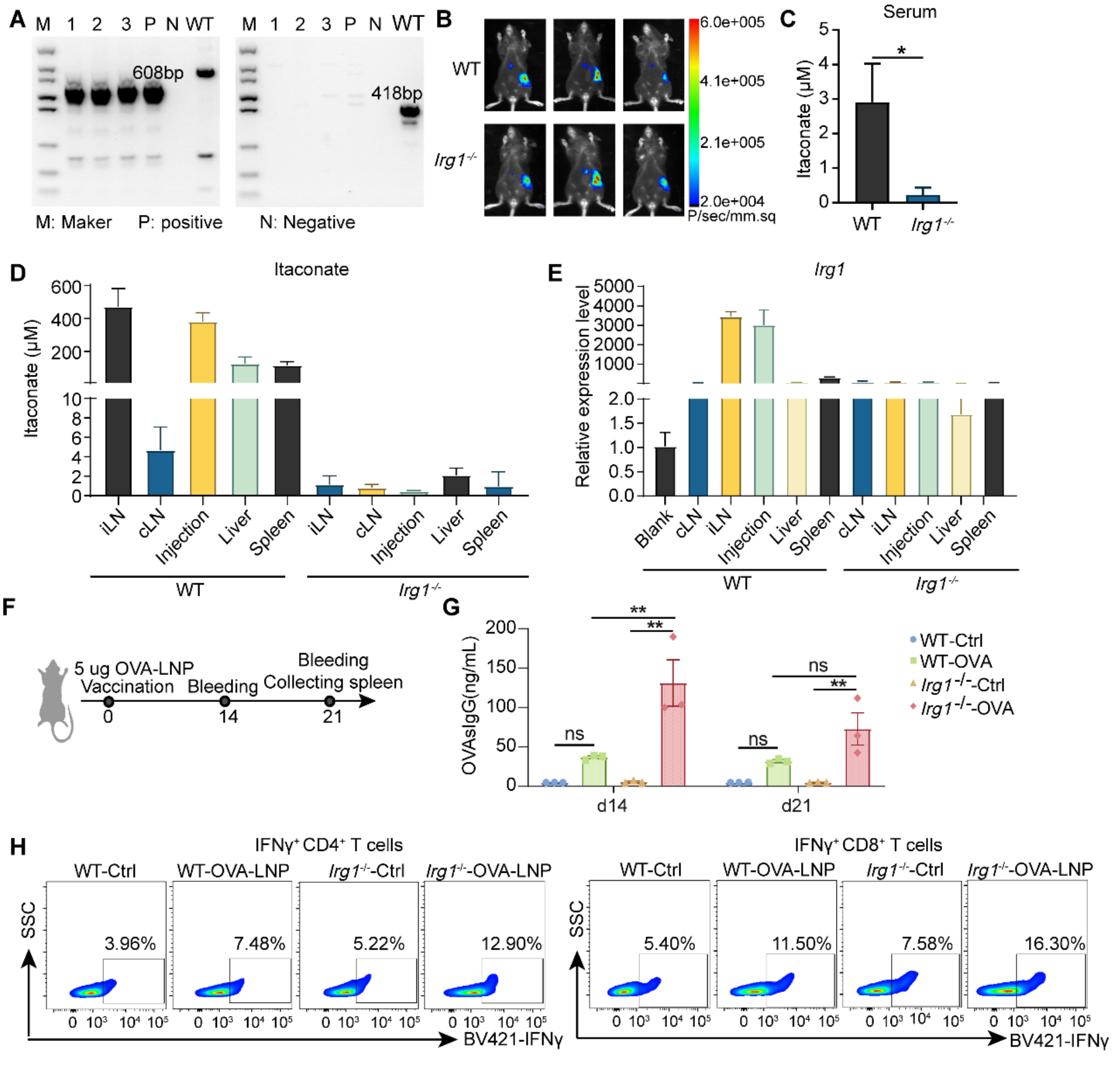

Figure S2. OVA-LNP-induced itaconate in iLNs suppresses T cell function

(A) The DNA agarose gel electrophoresis of WT and *Irg1*$^{-/-}$ mice. (B) The luminescence image of WT and *Irg1*$^{-/-}$ mice after Luciferase-LNP injection for 24h. (C-D) The itaconate concentration in the blood (C) and organs (D) of WT and *Irg1*$^{-/-}$ mice after OVA-LNP injection for 24h, n = 3. (E) *Irg1* mRNA expression of organs in WT and *Irg1*$^{-/-}$ mice after OVA-LNP injection for 24h, n = 3. (F) The schematic diagram of the process in WT and *Irg1*$^{-/-}$ mice. (G) The concentration of OVA sIgG in blood serum was detected by ELISA, n = 3. (H) The representative flow cytometry images of IFNγ^{+} CD4^{+} (left) and IFNγ^{+} CD8^{+} (right) T cells. ns = no significance, * $p < 0.05$, ** $p < 0.01$, *** $p < 0.001$.

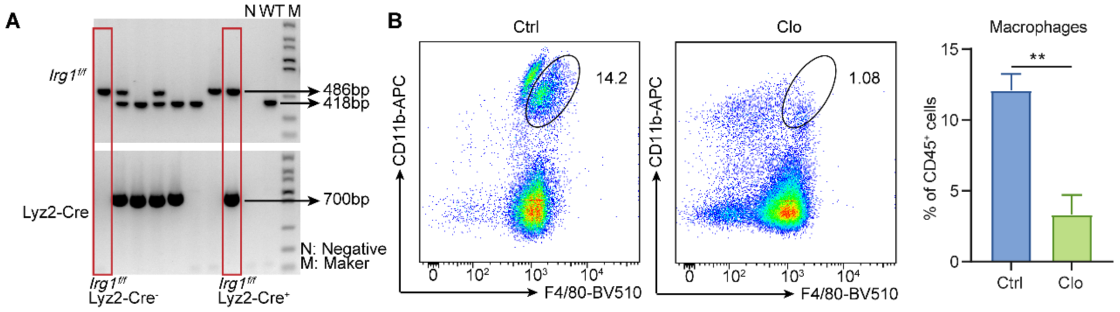

Figure S3. OVA-LNP-induced itaconate derives from macrophages. (A) DNA agarose gel electrophoresis of *Irg1*$^{f/f}$ Lyz2 $^{cre+}$ and *Irg1*$^{f/f}$ Lyz2 $^{cre-}$ mice. (B) The effectiveness of macrophages deletion in mice treated with 200 μL clodronate liposomes (Clo) after 24h detected by flow cytometry, n = 3. ns = no significance, * $p < 0.05$, ** $p < 0.01$, *** $p < 0.001$.

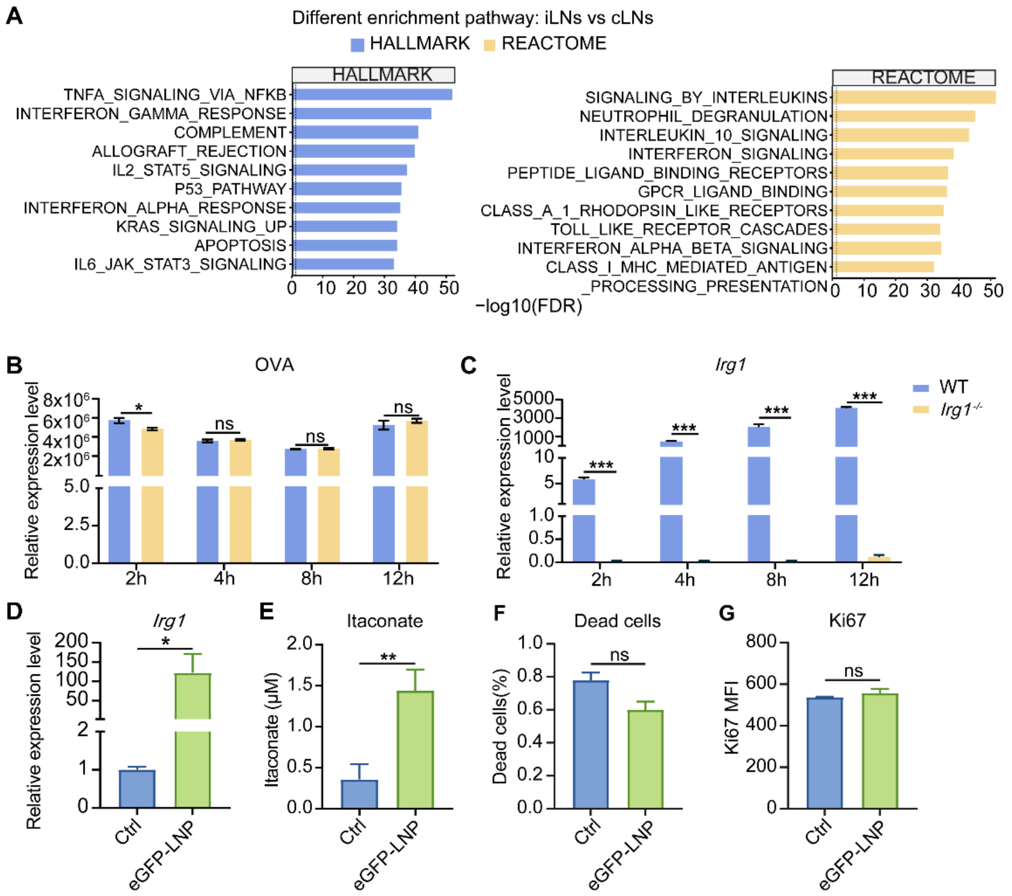

Figure S4. *Irg1*-induced by OVA-LNP suppressed the pro-inflammatory of macrophages.

(A) The HALLMARK and REACTOME enrichment of macrophages after OVA-LNP stimulation in iLNs compared to cLNs. (B-C) The OVA (B) and *Irg1* (C) expression levels of WT and *Irg1*$^{-/-}$ macrophages after 0.3 μg/mL OVA-LNP stimulation at different time points. (D) *Irg1* mRNA expression of BMDMs after 0.3 μg/mL eGFP-LNP stimulation for 12h. (E) The concentration of itaconate of BMDMs after 0.3 μg/mL eGFP-LNP stimulation for 24h. (F) 7-AAD-positive and (G) Ki67-positive BMDMs were detected by flow cytometry after 0.3 μg/mL eGFP-LNP stimulation for 24h. ns = no significance, * $p < 0.05$, ** $p < 0.01$, *** $p < 0.001$.

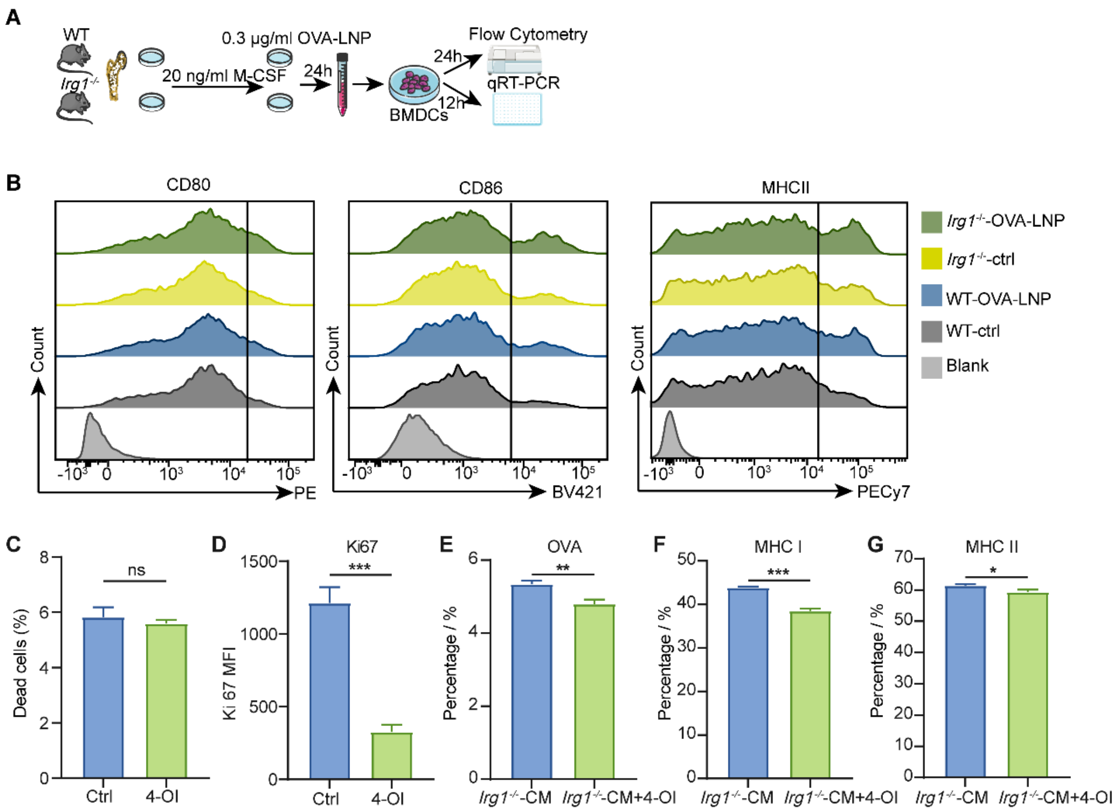

Figure S5. Itaconate suppressed the function of DC.

(A) The schematic diagram of the preparation of WT and *Irg1*$^{-/-}$ macrophage-derived CM. (B) The representative histogram of CD86, CD86, and MHC II of BMDC, cultured with WT and *Irg1*$^{-/-}$ macrophage-derived CM. (C) 7-AAD-positive and (D) Ki67-positive BMDCs were detected by flow cytometry after 125 μM 4-OI treatment for 24h. (E-G) The OVA (E), MHC I (F), and MHC II (G) expression of BMDCs were detected by flow cytometry after *Irg1* BMDMs-derived CM and 125 μM 4-OI treatment for 24h. ns = no significance, * $p < 0.05$, ** $p < 0.01$, *** $p < 0.001$.

A

Sample name	Encapsulation efficiency (≥80%)	Encapsulated mRNA concentration (≥ 0.100 mg/mL)
OVA-LNP	90%-95%	0.100-0.129
OVA&si-Irg1-LNP	90%-98%	0.100-0.141

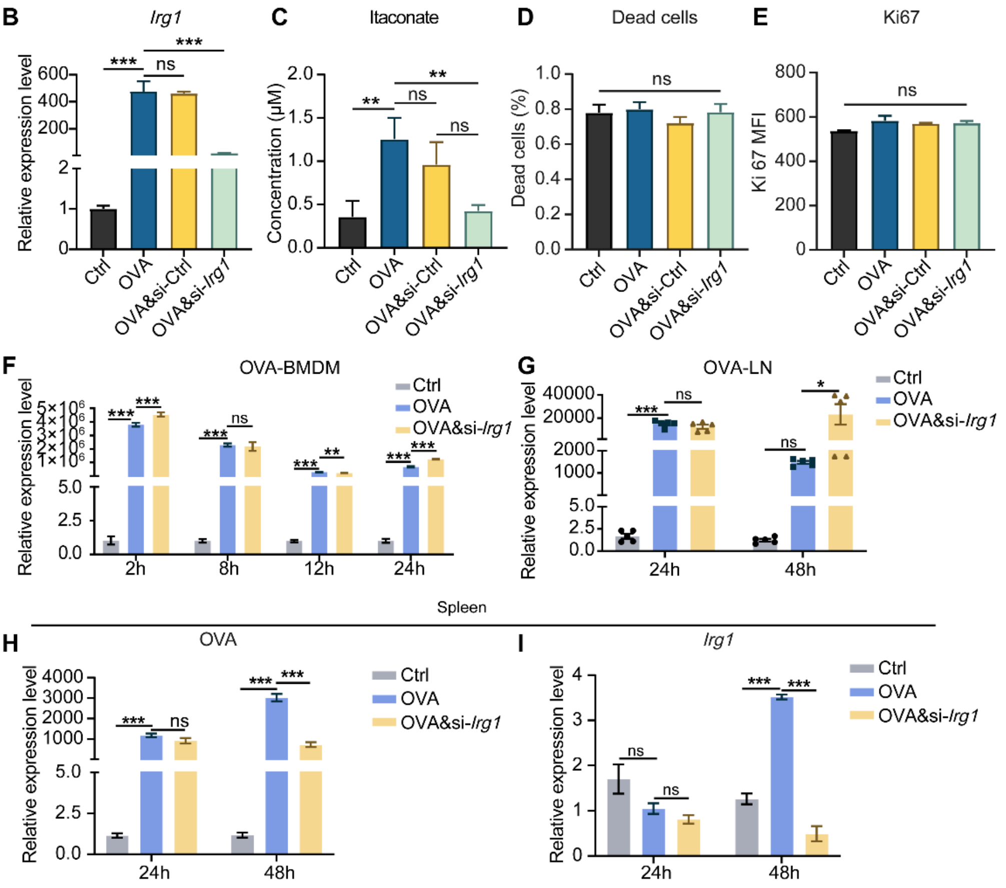

Figure S6. The OVA and *Irg1* expression induced by OVA-LNP and OVA&si- *Irg1*-LNP *in vitro* and *in vivo*.

(A) The encapsulation efficiency and encapsulated mRNA concentration of LNPs detected by the RiboGreen Kit. (B) *Irg1* mRNA expression of BMDMs after 0.3 μg/mL Ctrl, OVA-LNP, OVA&si-Ctrl-LNP, and OVA&si-*Irg1*-LNP stimulation for 12h. (C) The concentration of itaconate of BMDMs after 0.3 μg/mL Ctrl, OVA-LNP, OVA&si-Ctrl-LNP, and OVA&si-*Irg1*-LNP stimulation for 24h. (D) 7-AAD-positive and (E) Ki67-positive BMDMs were detected by flow cytometry after 0.3 μg/mL Ctrl, OVA-LNP, OVA&si-Ctrl-LNP, and OVA&si-*Irg1*-LNP stimulation for 24h. (F) OVA

expression levels of BMDMs after treatment with 0.3 μg/mL OVA-LNP and OVA&si-*Irg1*-LNP for different time points detected by qRT-PCR. (G) OVA expression levels of LNs after treatment with 5 μg OVA-LNP and OVA&si-*Irg1*-LNP subcutaneously for 24 and 48 h were detected by qRT-PCR, n = 5. (H-I) OVA (H) and *Irg1*(I) expression in spleens after stimulation with OVA-LNP and OVA&si- *Irg1*-LNP for 24 and 48 h.ns = no significance, * $p < 0.05$, ** $p < 0.01$, *** $p < 0.001$.

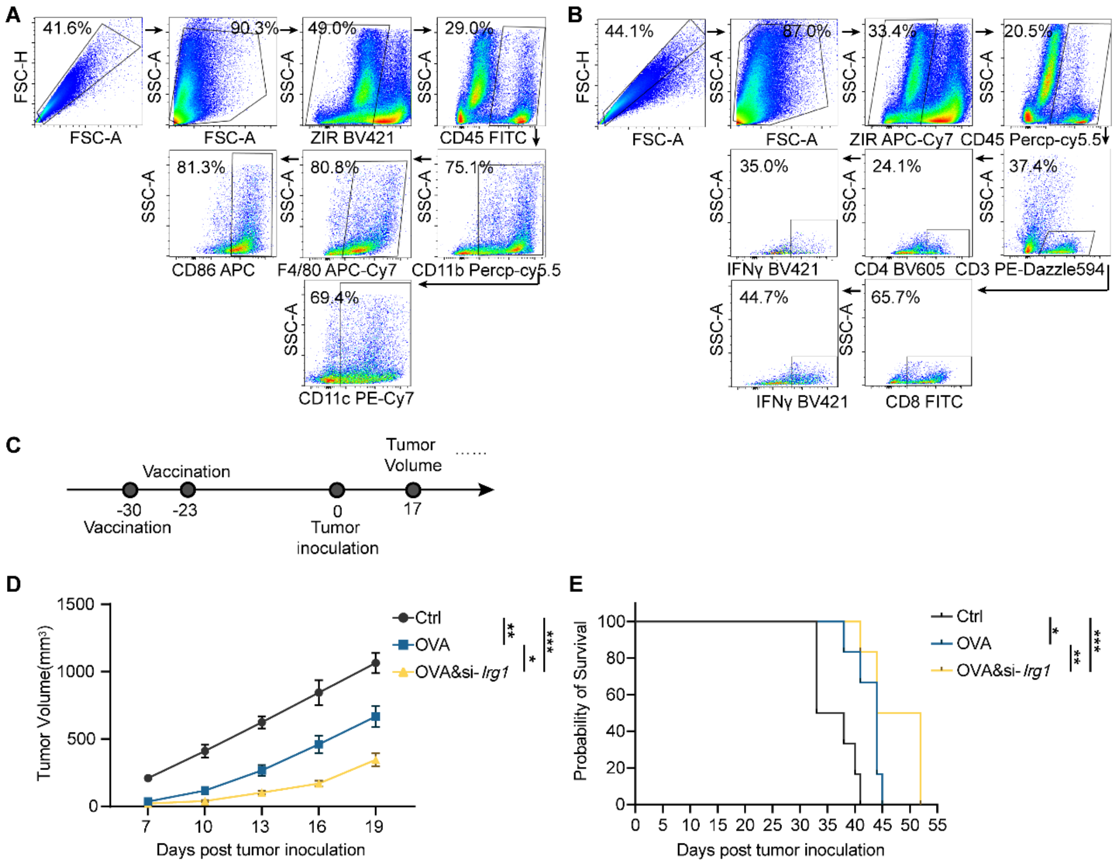

Figure S7. OVA&si-*Irg1*-LNP showed a protective effect in the B16-F10 melanoma mouse model.

(A-B) The gating strategy of the B16-F10-OVA melanoma mouse model administration with LNP. (A) The gating strategy of myeloid cells in the TME. (B) The gating strategy of lymphoid cells in the TME. (C) The schematic diagram of the B16-F10-OVA-beared melanoma mouse model, n = 5. (D) The tumor growth curve of the B16-F10-beared

mice before administration with two-dose LNPs. (E) Kaplan-Meier analysis of B16-F10-OVA melanoma mice. ns = no significance, * $p < 0.05$, ** $p < 0.01$, *** $p < 0.001$.

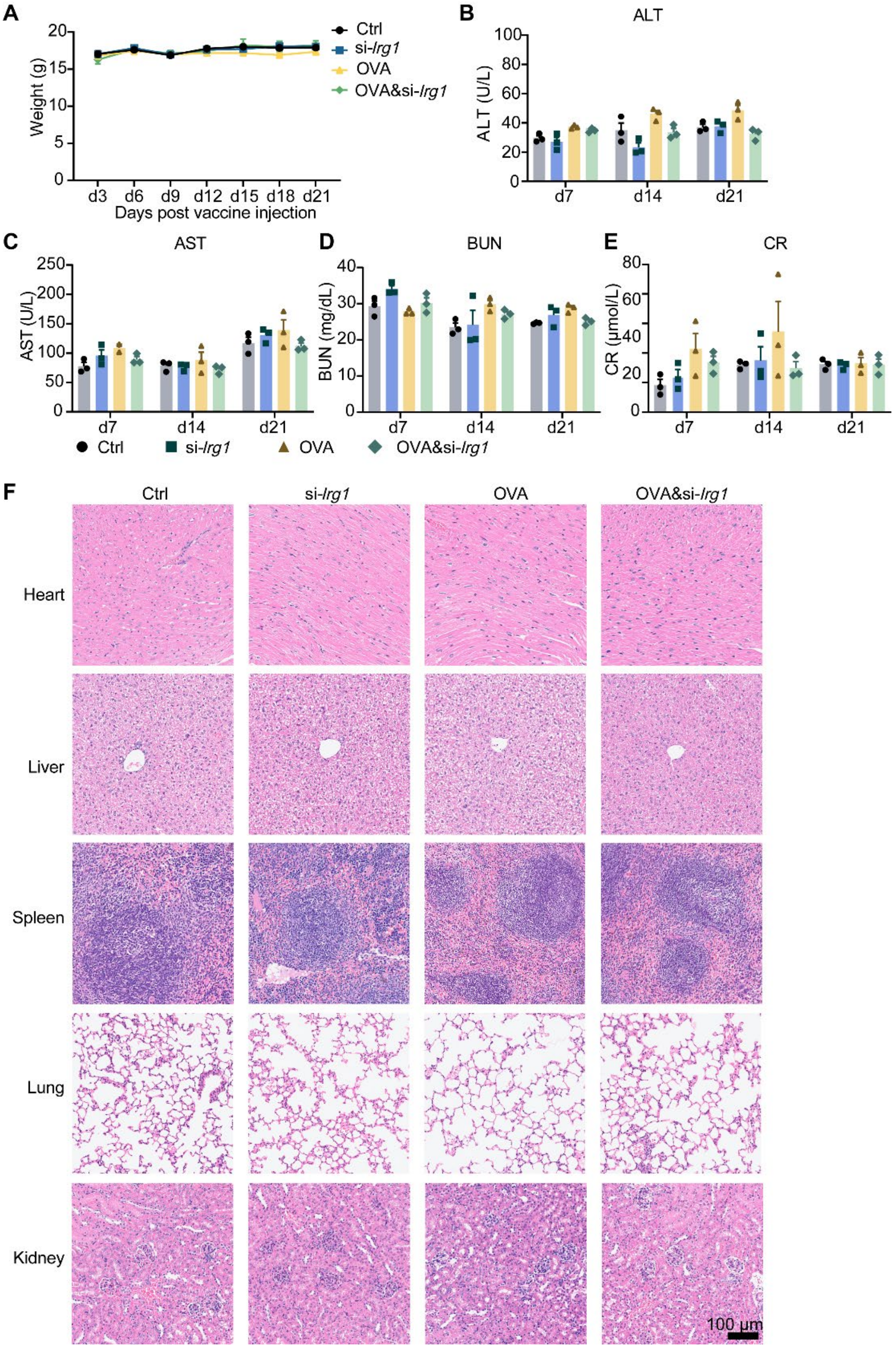

Figure S8. The safety of LNPs.

(A) Mice's weight was monitored every 3 days after 5 μg LNP injection subcutaneously,

n = 3. (B-E) The biochemical assay of ALT (B), AST (C), BUN (D), and CR (E) in the blood serum at days 7, 14, and 21. (F) H&E staining of heart, liver, spleen, lung, and kidney at day 21.

Supplementary Tables

Table S1. The antibodies in this study.

**Antibodies**	**Catalog Number**
PE anti-CD80	104708, Biolegend
Brilliant Violet 421^{TM} anti-CD86	105032, Biolegend
FITC anti-MHC I	116506, Biolegend
PE-Cyanine7 anti-MHC II	107614, Biolegend
APC anti-CCR7	120108, Biolegend
FITC anti-CD8	100706, Biolegend
PE anti-IFNγ	505808, Biolegend
PE/Cyanine7 anti-CD45	103114, Biolegend
PE/Dazzle^{TM} 594 anti-CD3	100246, Biolegend
Brilliant Violet 605^{TM} anti-CD4	116027, Biolegend
Brilliant Violet 421^{TM} anti-IFNγ	505830, Biolegend
APC anti-CD11b	101212, Biolegend
Brilliant Violet 605^{TM} anti-CD11c	117334, Biolegend
Brilliant Violet 510^{TM} anti-F4/80	123135, Biolegend

PerCP/Cyanine5 anti-CD19	1524406, Biolegend
PE anti-NK-1.1	156504, Biolegend
PerCP/Cyanine5.5 anti-CD45	157612, Biolegend
FITC anti-CD3	100203, Biolegend
PE anti-CD4	100408, Biolegend
Brilliant Violet 510^{TM} anti-CD8	100752, Biolegend
PE anti-Granzyme B	372208, Biolegend
PE anti-CD11b	101208, Biolegend
Brilliant Violet 421^{TM} anti-CD86	105032, Biolegend
APC anti-H-2K^b bound to SIINFEKL	141606, Biolegend
FITC anti-CD45	147710, Biolegend
PerCP/Cyanine5.5 anti-CD11b	101228, Biolegend
PE/Cyanine7 anti-CD11c	117318, Biolegend
APC/Cyanine7 anti-F4/80	123118, Biolegend
APC anti-CD86	159216, Biolegend
PE anti-CD206	141706, Biolegend

Supplementary Table 2. The primers in this study.

**Primer Name**	**Primer Sequence**
Gapdh Forward	CATCACTGCCACCCAGAAGACTG
Gapdh Reverse	ATGCCAGTGAGCTTCCCGTTCAG
OVA Forward	CCAGGACACAAATCAACAA

OVA Reverse	GGCAGAATAGGGTAACGCT
Irg1 Forward	AGTTTTCTGGCCTCGACCTG
Irg1 Reverse	AGAGGGAGGGTGGAATCTCT
Il1β Forward	TGGACCTTCCAGGATGAGGACA
Il1β Reverse	GTTCATCTCGGAGCCTGTAGTG
Il6 Forward	TACCACTTCACAAGTCGGAGGC
Il6 Reverse	CTGCAAGTGCATCATCGTTGTTC
Il8 Forward	GGTGATATTCGAGACCATTTACTG
Il8 Reverse	GCCAACAGTAGCCTTCACCCAT
Il23α Forward	CATGCTAGCCTGGAACGCACAT
Il23α Reverse	ACTGGCTGTTGTCCTTGAGTCC
Cxcl9 Forward	CCTAGTGATAAGGAATGCACGATG
Cxcl9 Reverse	CTAGGCAGGTTTGATCTCCGTTC
Cxcl10 Forward	ATCATCCCTGCGAGCCTATCCT
Cxcl10 Reverse	GACCTTTTTTGGCTAAACGCTTTC
Cxcl11 Forward	CCGAGTAACGGCTGCGACAAAG
Cxcl11 Reverse	CCTGCATTATGAGGCGAGCTTG
Ccr7 Forward	AGAGGCTCAAGACCATGACGG
Ccr7 Reverse	TCCAGGACTTGGCTTCGCTGTA
